# Circuit and Cellular Mechanisms Facilitate the Transformation from Dense to Sparse Coding in the Insect Olfactory System

**DOI:** 10.1523/ENEURO.0305-18.2020

**Published:** 2020-03-27

**Authors:** Rinaldo Betkiewicz, Benjamin Lindner, Martin P. Nawrot

**Affiliations:** 1 Bernstein Center for Computational Neuroscience Berlin, 10115 Berlin, Germany; 2 Computational Systems Neuroscience, Institute of Zoology, University of Cologne, 50674 Cologne, Germany; 3 Department of Physics, Humboldt University Berlin, 12489 Berlin, Germany

**Keywords:** efficient coding, lateral inhibition, odor trace, sensory processing, spike frequency adaptation, spiking neural network

## Abstract

Transformations between sensory representations are shaped by neural mechanisms at the cellular and the circuit level. In the insect olfactory system, the encoding of odor information undergoes a transition from a dense spatiotemporal population code in the antennal lobe to a sparse code in the mushroom body. However, the exact mechanisms shaping odor representations and their role in sensory processing are incompletely identified. Here, we investigate the transformation from dense to sparse odor representations in a spiking model of the insect olfactory system, focusing on two ubiquitous neural mechanisms: spike frequency adaptation at the cellular level and lateral inhibition at the circuit level. We find that cellular adaptation is essential for sparse representations in time (temporal sparseness), while lateral inhibition regulates sparseness in the neuronal space (population sparseness). The interplay of both mechanisms shapes spatiotemporal odor representations, which are optimized for the discrimination of odors during stimulus onset and offset. Response pattern correlation across different stimuli showed a nonmonotonic dependence on the strength of lateral inhibition with an optimum at intermediate levels, which is explained by two counteracting mechanisms. In addition, we find that odor identity is stored on a prolonged timescale in the adaptation levels but not in the spiking activity of the principal cells of the mushroom body, providing a testable hypothesis for the location of the so-called odor trace.

## Visual Abstract

**Figure:**
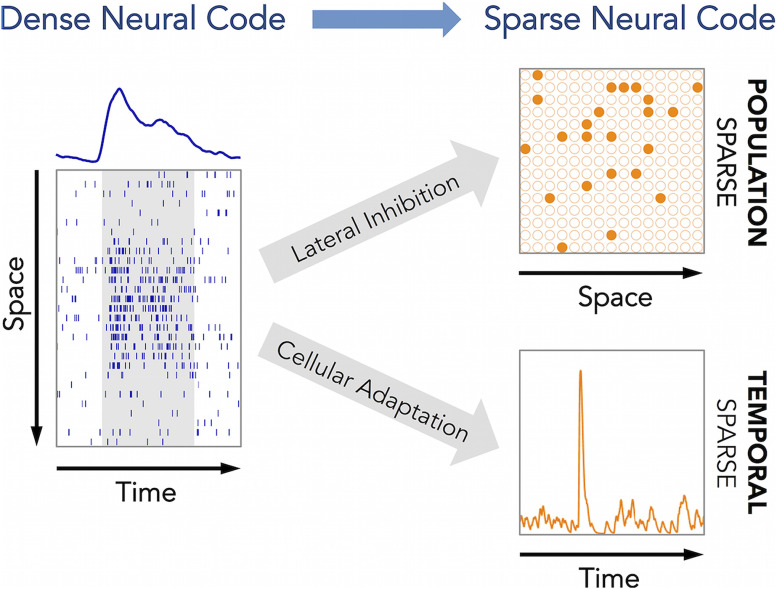

## Significance Statement

In trace conditioning experiments, insects, like vertebrates, are able to form an associative memory between an olfactory stimulus and a temporally separated reward. Forming this association requires a prolonged odor trace. However, spiking responses in the mushroom body, the principal site of olfactory learning, are brief and bound to the odor onset (temporal sparseness). We implemented a spiking network model that relies on spike frequency adaptation to reproduce temporally sparse responses. We found that odor identity is reliably encoded in neuron adaptation levels, which are mediated by spike-triggered calcium influx. Our results suggest that a prolonged odor trace is established in the calcium levels of the relevant neuronal population. This prediction has found recent experimental support in the fruit fly.

## Introduction

How nervous systems process sensory information is a key issue in systems neuroscience. Animals are required to rapidly identify behaviorally relevant stimulus features in a rich and dynamic sensory environment, and neural computation in sensory pathways is tailored to this need. Sparse stimulus encoding has been identified as an essential feature of sensory processing in higher brain areas in both invertebrate (Perez-Orive et al., 2002; Szyszka et al., 2005; Ito et al., 2008; Turner et al., 2008; Honegger et al., 2011) and vertebrate (Vinje and Gallant, 2000; Hromádka et al., 2008; Isaacson, 2010; Wolfe et al., 2010) systems. Sparse representations provide an economical means of neural information coding (Laughlin and Sejnowski, 2003; Faisal et al., 2008) where information is represented by only a small fraction of all neurons (population sparseness) and each activated neuron generates only few action potentials (temporal sparseness) for a highly specific stimulus configuration (lifetime sparseness; Kloppenburg and Nawrot, 2014).

The nervous systems of insects have limited neuronal resources and thus require particularly efficient coding strategies. The insect olfactory system is analogue to the vertebrate olfactory system and has become a popular model system for the emergence of a sparse code. We use a computational approach to study the transformation from a dense olfactory code in the sensory periphery to a sparse code in the mushroom body (MB), a central structure of the insect brain important for multimodal sensory integration and memory formation. A number of recent studies emphasized the role of sparse coding in the MB. In locusts, sparse responses were shown to convey temporal stimulus information (Gupta and Stopfer, 2012). In *Drosophila*, sparse coding was found to reduce overlap between odor representations and facilitate their discrimination (Lin et al., 2014). Consequently, sparse coding is an essential feature of plasticity models for olfactory learning in insects (Huerta and Nowotny, 2009; Wessnitzer et al., 2012; Ardin et al., 2016; Peng and Chittka, 2016; Müller et al., 2018), and theoretical work has emphasized the analogy of the transformation from a dense code in projection neurons (PNs) to a sparse code in Kenyon cells (KCs) with dimensionality expansion in machine learning methods (Huerta and Nowotny, 2009; Mosqueiro and Huerta, 2014; Schmuker et al., 2014).

Central to our modeling approach are two fundamental mechanisms of neural computation that are ubiquitous in the nervous systems of invertebrates and vertebrates. Spike frequency adaptation (SFA) is a cellular mechanism that has been suggested to support efficient and sparse coding and to reduce the variability of sensory representation (Benda and Herz, 2003; Farkhooi et al., 2011, 2013). Lateral inhibition is a basic circuit design principle that exists in different sensory systems, mediates contrast enhancement, and facilitates stimulus discrimination (Kuffler, 1953; Hartline et al., 1956; Fuchs and Brown, 1984; Oswald et al., 2006). Both mechanisms are evident in the insect olfactory system. Responses of olfactory receptor neurons (ORNs), local interneurons (LNs), and PNs in the antennal lobe (AL) show stimulus adaptation (Bhandawat et al., 2007; Krofczik et al., 2009; Nagel and Wilson, 2011), and strong adaptation currents have been identified in KCs (Wüstenberg et al., 2004; Demmer and Kloppenburg, 2009). Lateral inhibition in the AL is mediated by inhibitory LNs (Wilson, 2013). It is crucial for establishing the population code at the level of PNs (Wilson et al., 2004; Krofczik et al., 2009; Olsen et al., 2010), for gain control (Stopfer et al., 2003; Olsen and Wilson, 2008), for decorrelation of odor representations (Wilson and Laurent, 2005), and for mixture interactions (Krofczik et al., 2009; Deisig et al., 2010; Capurro et al., 2012).

Together, we find that lateral inhibition and SFA account for the transformation from a dense to sparse coding, decorrelate odor representations, and facilitate precise temporal responses on short and long timescales.

## Materials and Methods

### Spiking network model

**Figure 1.**
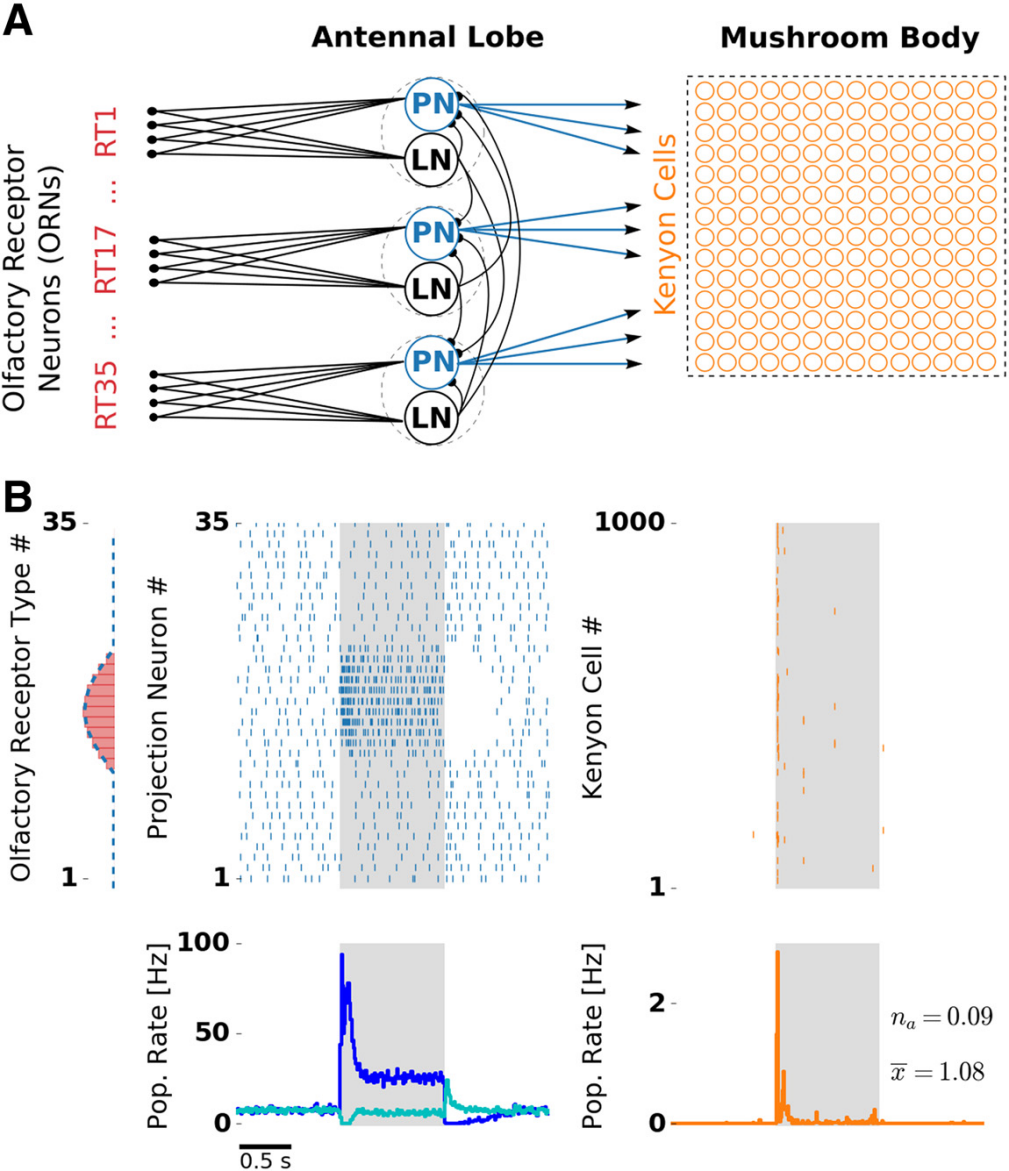
Olfactory network model structure and odor response. ***A***, Network structure resembles the insect olfactory pathway with three main processing stages. In each glomerulus (dashed circles), a PN (blue) and a LN receive convergent ORN input (red) by one receptor type (RT). Each LN provides unspecific lateral inhibition to all PNs. KCs (orange) receive on average 12 inputs from randomly chosen PNs. ***B***, Receptor response profile (red bars, AL input) depicts the evoked firing rate for each ORN type. Evoked PN spike counts (dashed blue line, AL output) follow the ORN activation pattern. Raster plots depict single-trial responses of PNs (blue) and KCs (orange). Presentation of an odor during 1000 ms is indicated by the shaded area. Population (Pop.) firing rates were obtained by averaging over 50 trials. PN spikes display a temporal structure that includes evoked transient responses at stimulus onset and offset, and a pronounced inhibitory postodor response. The PN population rate was averaged over PNs showing on responses (blue) and off responses (cyan). KC spikes were temporally sparse with the majority of the spikes occurring at the stimulus onset. Extended Data Figure 1-1 and Extended Data Figure 1-2 show odor responses with adaptation disabled in the KC and PN population, respectively.

A spiking network model with three layers (ORN, AL, and MB; Fig. 1*A*) was simulated using Brian 1.4 (Goodman and Brette, 2009). The model includes 35 ORN types, 284 ORNs per type, 35 PNs and LNs, and 1000 KCs. Each of the 35 LN–PN pairs constitutes a glomerulus. Across insect species, the number of glomeruli varies from a few tens to several hundred, and we based our model on the lower end of this range. The ratio between the numbers of PNs and KCs is approximately based on the data available in *Drosophila* (Turner et al., 2008).

10.1523/ENEURO.0305-18.2020.f1-1 Figure 1-1 Odor response with selective adaptation in the LN and the PN population. Strong phasic PN input elicits phasic KC responses. High KC firing threshold ensures sparse responses in the absence of SFA in the KC population. Download Figure 1-1, TIF file.

**Figure 2.**
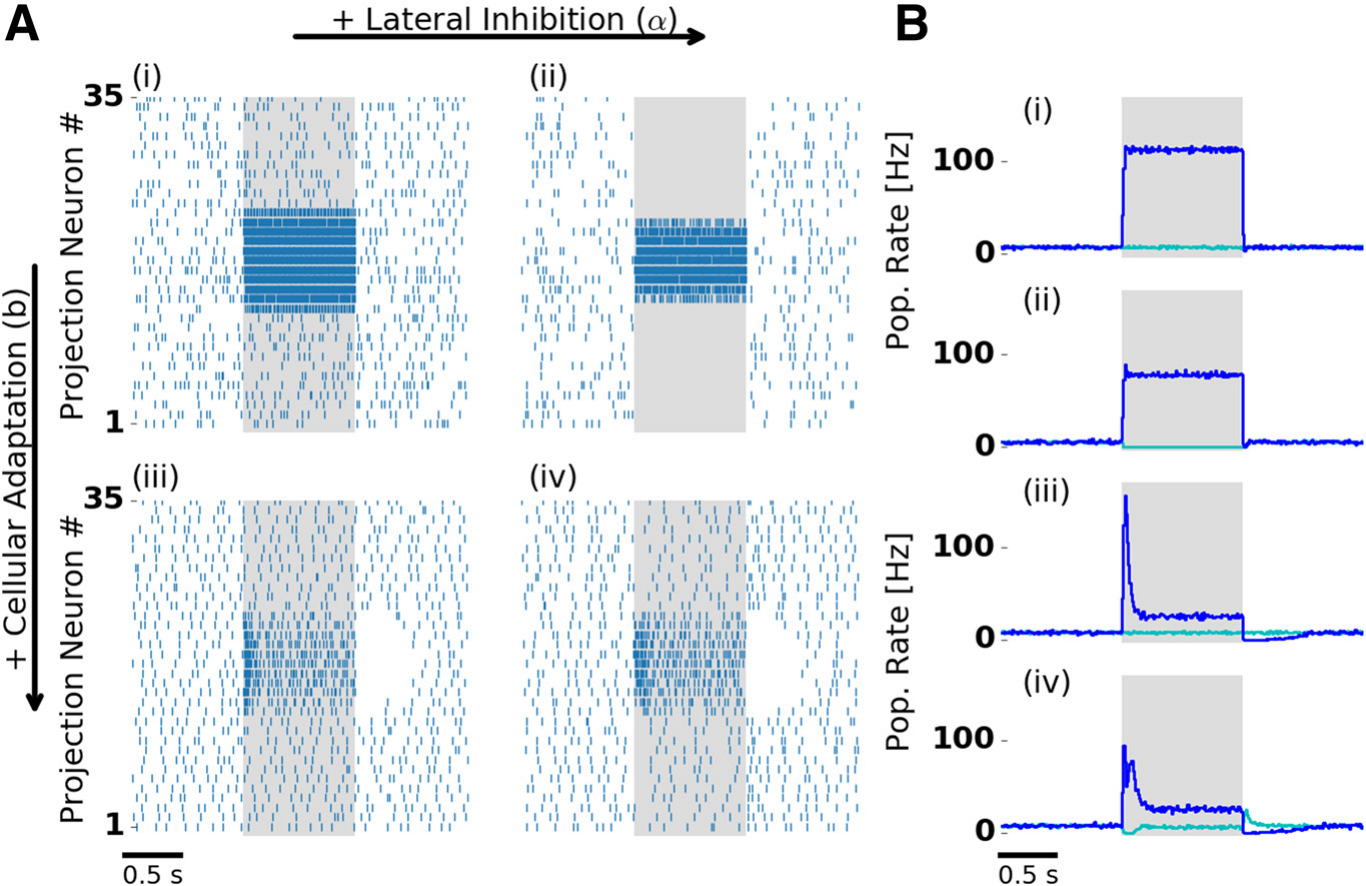
Lateral inhibition and cellular adaptation shape PN odor response dynamics. ***A***, Single-trial PN spiking responses simulated with (right column) and without (left column) lateral inhibition, and with (bottom row) and without (top row) adaptation. Presentation of a single odor during 1000 ms is indicated by the shaded area. With adaptation, PNs display a temporal structure that includes a transient and a tonic response, and a pronounced inhibitory postodor response. ***B***, Trial-averaged population firing rate: PNs driven by stimulation (blue) and remaining PNs (cyan). ***Bi–iv*** indicate the presence and absence of lateral inhibition and adaptation, as in ***A***. In the presence of lateral inhibition, firing rates during stimulation are reduced. In the presence of lateral inhibition and adaptation (***Aiv***, ***Biv***) PNs show either transient on responses (blue) or off responses (cyan). ***Aiv*** and ***Biv*** are reproduced in Figure 1*B*. Extended Data Figure 2-1 shows PN tuning profiles and input–output relation.

10.1523/ENEURO.0305-18.2020.f1-2 Figure 1-2 Odor response with selective adaptation in the LN and the KC population. The absence of SFA in the PN population was compensated by a constant current *I*_0_ = 0.38 nA. PNs show a constant population rate response with a slightly delayed onset due to inhibition by LNs. KCs show a strong onset population rate response and a nonzero tonic firing rate. Download Figure 1-2, TIF file.

The connections between the three network layers (ORNs, AL, MB) are feedforward and excitatory. Within the AL, LNs provide lateral inhibition to PNs. ORNs provide input to PNs and LNs. All ORNs of the same receptor type target the same single glomerulus. Every LN has inhibitory connections with all PNs, mediating unspecific lateral inhibition within the AL. Every KC receives 12 PN inputs on average (Szyszka et al., 2005; Turner et al., 2008). Connections between PNs and KCs were randomly drawn. Synaptic weights between all neurons are given in Table 1 for four different simulation conditions.

**Table 1:** Synaptic weights for *w_OL_* (ORN-LN), *w_OP_* (ORN-PN), *w_LP_* (LN-PN), and *w_PK_* (PN-KC) connections in different simulation conditions (i–iv)

	i	ii	iii	iv
*w*_OL_	1 nS	1 nS	1 nS	1 nS
*w*_OP_	1 nS	1.12 nS	1 nS	1.12 nS
*w*_LP_	0 nS	3 nS	0 nS	3 nS
*w*_PK_	5 nS	5 nS	5 nS	5 nS

Responses to a set of seven stimuli, 50 trials each, and 3000 ms trial duration were simulated. Stimuli had a duration of 1000 ms and were presented at *t* = 1000 ms. All neurons were initialized with membrane voltage set to the leak potential and the adaptation current set to zero. To achieve steady-state conditions, simulations were prerun for 2000 ms without recording the activity.

### Receptor input

ORNs were modeled as Poisson spike generators, with evoked firing determined by a receptor response profile and a spontaneous baseline. In the absence of stimulus, the spontaneous firing rate of all ORNs is set to $r_{O}^{BG}=20\;\text{Hz}$. In the presence of a stimulus, the ORN firing rate is given by the summation of the spontaneous rate and an activation $\Delta r_{O}$, as follows:

(1) $$r_{O}\left(t\right)=\{\begin{array}{ll} r_{O}^{BG}+\Delta r_{O} & \;\text{for}\;{\text{t}}_{\text{start}}<\text{t}<{\text{t}}_{\text{stop}}\\ r_{O}^{BG} & \text{else} \end{array}.$$

The intensity (amplitude) of ORN activation, $\Delta r_{O}$, is given by the receptor response profile that depends on receptor type and stimulus identity. Receptor activation follows a sine profile over half a period (0...π), as follows:

$$\Delta r_{O}=40\;\text{Hz}\;\{\begin{array}{ll} \sin\left(x\pi \right) & \text{for}\;\text{0}<\text{x}<\text{1}\\ 0 & \text{else} \end{array},$$

$$x=\frac{\left(k_{RT}-k_{S}\right)\;\mathrm{mod}\;N_{RT}}{N_{A}+1}\text{,}$$

where *k_S_* is the stimulus index, *k*_RT_ is the receptor type index, *N*_RT_ = 35 is the total number of receptor types and *N_a_* = 11 is the number of receptor types activated by a stimulus. Given these parameters, 35 different odor responses can be simulated ($k_{S}=0...34$). This profile ensures that odor responses are evenly distributed across receptor types, while the choice of the sine shape was arbitrary. If the difference between the index of two stimuli Δ*k_s_* is small, those two stimuli are called similar, because they elicit largely overlapping responses. For $\Delta k_{s}>12$, the responses do not overlap, representing dissimilar stimuli.

### Neuron model

PNs, LNs, and KCs were modeled as leaky integrate-and-fire neurons with conductance-based synapses and a spike-triggered adaptation (Treves, 1993) current *I^A^*. We use the same set of cell parameters for all neuron types (Table 2). This supports the generic character of our model and ensures that effects reported in this study are not a result of neuron type-specific parameters. The membrane potential of the *i*th neuron from the PN, LN, and KC populations obeys the following:

(2) $$c_{m}\frac{d}{dt}v_{i}^{P}=g_{L}\left(E_{L}-v_{i}^{P}\right)\;+\;g_{i}^{OP}\left(E_{E}-v_{i}^{P}\right)\;+\;g^{LP}\left(E_{I}-v_{i}^{P}\right)-I_{i}^{A}\text{,}$$

(3) $$c_{m}\frac{d}{dt}v_{i}^{L}=g_{L}\left(E_{L}-v_{i}^{L}\right)\;+\;g_{i}^{OL}\left(E_{E}-v_{i}^{L}\right)-I_{i}^{A}\text{,}$$

(4) $$c_{m}\frac{d}{dt}v_{i}^{K}=g_{L}\left(E_{L}-v_{i}^{K}\right)\;+\;g_{i}^{PK}\left(E_{E}-v_{i}^{K}\right)-I_{i}^{A}.$$

**Table 2:** Parameters of the neuron model

Neuron parameters		
Membrane capacitance	*c_m_*	289.5 pF
Leak conductance	*g_L_*	28.95 nS
Leak potential	*E_L_*	−70 mV
Reset potential	*V_R_*	−70 mV
Threshold potential	*V_T_*	−57 mV
Refractory time	*τ*_ref_	5 ms
Synaptic parameters		
Base synaptic weight	*w*_0_	1 nS
PN-KC synaptic weight	*w_PK_*	5 nS
Excitatory synaptic potential	*E_E_*	0 mV
Excitatory time constant	*τ_E_*	2 ms
Inhibitory synaptic potential	*E_I_*	−75 mV
Inhibitory time constant	*τ_I_*	10 ms
Adaptation parameters		
Spike-triggered current	$\Delta I^{A}$	0.132 nA
Adaptation time constant	*τ_A_*	389 ms
Adaptation current variance	$\sigma_{I}^{2}$	87.1 p*A*^2^

Membrane potentials follow a fire-and-reset rule. The fire-and-reset rule defines the spike trains of PNs, LNs, and KCs denoted by $x_{i}^{B}=\sum_{k}\delta \left(t-t_{ik}^{B}\right)$ for the *i*th neuron of type B. The spike trains of the ORN neurons are generated by a Poisson process with spike times $t_{ijk}^{O}$ for the *j*th receptor neuron of the *k*th receptor type:

(5) $$x_{i}^{O}\left(t\right)=\sum_{j}^{N_{O}/N_{glu}}\sum_{k}^{N_{glu}}\delta \left(t-t_{ijk}^{O}\right).$$

Synaptic conductances *g_i_* obey the following:

(6) $$\tau_{E}\frac{d}{dt}g_{i}^{OP}=-g_{i}^{OP}\;+\;\tau_{E}w_{OP}x_{i}^{O}\left(t\right)\text{,}$$

(7) $$\tau_{E}\frac{d}{dt}g_{i}^{OL}=-g_{i}^{OL}\;+\;\tau_{E}w_{OL}x_{i}^{O}\left(t\right)\text{,}$$

(8) $$\tau_{I}\frac{d}{dt}g^{LP}=-g^{LP}\;+\;\tau_{I}w_{LP}\sum_{j}^{N_{Glu}}x_{j}^{L}\left(t\right)\text{,}$$

(9) $$\tau_{E}\frac{d}{dt}g_{i}^{PK}=-g_{i}^{PK}\;+\;\tau_{E}\sum_{j}^{N_{Glu}}W_{ij}x_{i}^{P}\left(t\right).$$

Adaptation currents $I_{i}^{A}$ obey the following:

(10) $$\tau_{A}\frac{d}{dt}I_{i}^{A}=-I_{i}^{A}\;+\;\tau_{A}\Delta I^{A}x_{i}\left(t\right)\;+\;\sqrt{2\tau_{A}\sigma_{I}^{2}}\xi \left(t\right).$$

where *τ_A_* is the time constant and Δ*I^A^* is the spike-triggered increase of the adaptation current. This phenomenological model of spike-triggered adaptation is biologically motivated by calcium-dependent outward potassium currents. Each action potential leads to an influx of a fixed amount of calcium, and intracellular calcium is removed only slowly, resulting in an exponential decay of the intracellular calcium level. The last term reflects the diffusion approximation of channel noise (Schwalger et al., 2010), where ξ(t) represents Gaussian white noise. The variance of the adaptation currents $I_{i}^{A}$ is given by $\sigma_{I}^{2}$.

### Simulation conditions

The following four different scenarios were simulated: (i) without lateral inhibition and cellular adaptation, (ii) with lateral inhibition, (iii) with cellular adaptation, and (iv) with lateral inhibition and cellular adaptation. We quantified the strength of lateral inhibition with a multiplicative factor, α, that sets the synaptic weight between LNs and PNs (w_LP_) in units of the base synaptic weight (w_0_), as follows:

(11) $$w_{LP}=\alpha w_{0}.$$

Lateral inhibition is a network effect, conveyed by synaptic transmission, and was therefore compensated by the scaling of synaptic weights. Weight scaling provides compensation during spontaneous as well as evoked activity. The scenario without lateral inhibition acts as a control condition, which deliberately does not include slow inhibitory synaptic dynamics.

In scenarios without cellular adaptation (1 and 2 above), the dynamic adaptation current was replaced by a compensatory static current, $I_{i}^{A}\equiv I_{0}=0.38\;\text{nA}$, in the PN and LN populations, whereas in the KC population it was set to zero, $I_{i}^{A}\equiv 0\;\text{nA}$. In scenarios without lateral inhibition (1 and 3 above) the inhibitory weights *w_LP_* were set to zero by setting *α* = 0. The synaptic weight *w_OL_* was adjusted to achieve a spontaneous LN firing rate of ∼8 Hz that is well within the experimentally observed range (Perez-Orive et al., 2002; Krofczik et al., 2009; Chou et al., 2010).

In all scenarios, the spontaneous firing rate of PNs was set to ∼8 Hz (Perez-Orive et al., 2002; Krofczik et al., 2009; Chou et al., 2010; Meyer et al., 2013) by adjusting the synaptic weights between the ORNs and the PNs (*w_OP_*).

### Code accessibility

Script files for model simulation are accessible at: https://github.com/nawrotlab/SparseCodingInSpikingInsectModel.

Running the simulation requires Python 2.7, Brian 1.4, and numpy 1.11. All code was run on a x86-64 Linux machine.

run_IF.py, run_saIF.py - simulation scripts were used to run the model in the absence and presence of SFA, respectively. All parameters are contained within the respective scripts. Running the script file will save simulation results to file in the Python pickle format.

sim_code.py is the code of the neuron, input, and network models.

### Data analysis

#### Population firing rate

The spike count of the *i*th neuron, in the *k*th time bin with size Δ*t* is given by the following:

(12) $$n_{i,k}=\int_{(k-1)\Delta t}^{k\Delta t}{\mathrm{dtx}}_{i}(t).$$

Population firing rates were obtained from the spike count in a small time bin (Δ*t *=* *10 ms), as follows:

$$\rho_{k}=\frac{1}{\Delta t}{〈n_{i,k}〉}_{i}\text{,}$$

where ${〈.〉}_{i}$ indicates the population average. In addition, population firing rates were averaged over 50 trials.

#### Sparseness measure

The sparseness of evoked KC responses was quantified by the widely used modified Treves–Rolls measure (Treves and Rolls, 1991; Willmore and Tolhurst, 2001):

$$s=1-\frac{{\left(\frac{1}{N}\sum_{i=1}^{N}a_{i}\right)}^{2}}{\frac{1}{N}\sum_{i=1}^{N}a_{i}^{2}}\text{,}$$

where *a_i_* indicates either the distribution of KC spike counts (population sparseness, for *i* between 1 and 1000), or binned KC population firing rate (temporal sparseness, Δ*t * = * *50 * *m*s*, for *i* between 1 and 20). The sparseness measure takes values between 0 and 1, and high values indicate sparse responses. Both measures were averaged over 50 trials.

#### Pattern overlap

We define the activation pattern for a given odor by a vector containing the evoked spike count for every neuron in a population. Pattern overlap between two similar odors, A and B, was calculated using an expression formally equivalent to Pearson’s correlation coefficient, as follows:

(13) $$\varrho_{AB,k}=\frac{N_{pop}\sum_{i}n_{ik}m_{ik}-\sum_{i}n_{ik}\sum_{j}m_{ik}}{\sqrt{N_{pop}\sum_{i}n_{ik}^{2}-{\left(\sum_{i}n_{ik}\right)}^{2}}\sqrt{N_{pop}\sum_{i}m_{ik}^{2}-{\left(\sum_{i}m_{ik}\right)}^{2}}}\text{,}$$

where *n_ik_* and *m_ik_* are the spike counts of the *i*th neuron, *k*th trial, in response to odor A and odor B (Δ*k_S_* = 2) respectively, and *N*_pop_ is the number of neurons in the population. The correlation coefficient was calculated both for the PN and the KC population, and was averaged over 50 trials and five network realizations with randomly drawn PN–KC connectivity.

In addition, we consider trial-averaged activation patterns ${\hat{n}}_{i}=\frac{1}{N_{trial}}\sum_{k}n_{ik}$ and ${\hat{m}}_{i}=\frac{1}{N_{trial}}\sum_{k}m_{ik}$. Based on these trial-averaged patterns, the overlap between those patterns is given by the following expression:

(14) $${\tilde{\varrho }}_{AB}=\frac{N_{pop}\sum_{i}{\hat{n}}_{i}{\hat{m}}_{i}-\sum_{i}{\hat{n}}_{i}\sum_{j}{\hat{n}}_{j}}{\sqrt{N_{pop}\sum_{i}{\hat{n}}_{i}^{2}-{\left(\sum_{i}{\hat{n}}_{i}\right)}^{2}}\sqrt{N_{pop}\sum_{i}{\hat{m}}_{i}^{2}-{\left(\sum_{i}{\hat{m}}_{i}\right)}^{2}}}.$$

The overlap between the trial-averaged patterns was calculated both for the PN and the KC population, and averaged over five network realizations with randomly drawn PN–KC connectivity.

#### Lateral inhibition scaling with parameter *α*

To test whether the decrease of overlap was robust for different strengths of lateral inhibition, the synaptic weight *w_OP_* was adjusted as follows:

(15) $$w_{OP}=w_{0}\left(1+\alpha b\right)\text{,}$$

where *b* was estimated from simulations under the condition that for a range of lateral inhibition strengths (α∈[0,9]) the spontaneous PN firing rate was close to 8 Hz.

### Decoding analysis

Odor identity was recovered from odor representations by Gaussian naive Bayes classification (Rish, 2000), using the scikit-learn package (Pedregosa et al., 2012). Training and testing data consisted of simulated odor representations for a set of seven stimuli ($k_{S}=0,2,...,12$), 50 trials each. Classification was repeated for every time bin (Δ*t * = * *50 ms, 60 bins total) for PN spike counts, KC spike counts, or amplitudes of KC adaptations currents. Data were divided into a training and testing set using a threefold cross-validation procedure. Decoding accuracy was estimated by the maximum *a posteriori* method and is given by the fraction of successful classification trials divided by the total number of test trials.

## Results

### Spiking network model of the olfactory pathway with lateral inhibition and spike frequency adaptation

We designed a spiking network model that reduces the complexity of the insect olfactory processing pathway to a simplified three-layer network (Fig. 1*A*) that expresses the structural commonality across different insect species, as follows: an input layer of ORNs, subdivided into different receptor types, the AL, a first-order olfactory processing center, and the MB. Furthermore, the model combines the following two essential computational elements: (1) lateral inhibition in the AL, and (2) spike frequency adaptation in the AL and the MB.

The processing between the layers is based on excitatory feedforward connections. Converging receptor input from all ORNs of one type is received by spatially confined subunits of the AL called glomeruli. In our model, glomeruli are represented by a single uniglomerular PN and a single inhibitory LN. In the MB, each KC receives, on average, 12 PN inputs (Szyszka et al., 2005), based on a random connectivity between the AL and the MB (Caron et al., 2013). All neurons in the AL and the MB were modeled as leaky integrate-and-fire neurons with spike-triggered adaptation. Based on evidence from theoretical studies (Schwalger et al., 2010) and experimental studies (Fisch et al., 2012), adaptation channels cause slow fluctuations. We accounted for this fact by simulating channel noise in the slow adaptation currents (Materials and Methods).

We simulated ORN responses to different odor stimuli. Single ORN responses were modeled in the form of Poisson spike trains with firing rates dependent on the receptor type and stimulus identity. The relationship is set by a receptor response profile (Fig. 1*B*, left) that determines ORN firing rates of all receptor types for a given stimulus. Responses to different stimuli are generated by shifting the response profile along the receptor space. The offset between any two stimuli reflects their dissimilarity: similar stimuli activate overlapping sets of olfactory receptors, whereas dissimilar stimuli activate largely disjoint sets of receptors. Stimuli were presented for 1 s, reflected by a step-like increase of ORN firing rate.

In the absence of stimuli, ORNs fired with a rate of 20 Hz reflecting their spontaneous activation (Nagel and Wilson, 2011). Both LNs and PNs receive direct ORN input. We tuned synaptic weights of the model to match physiologically observed firing rates of PNs and LNs, which are both ∼8 Hz (Perez-Orive et al., 2002; Krofczik et al., 2009; Chou et al., 2010; Meyer et al., 2013; for details, see Materials and Methods). Lateral inhibition and SFA, the neural mechanisms under investigation, both provide an inhibitory contribution to the total input of a neuron. In our model, SFA is a cellular mechanism mediated by a slow, spike-triggered, hyperpolarizing current in LNs, PNs, and KCs, whereas a global lateral inhibition in the AL is mediated by LNs with fast synapses that receive input from a single ORN type and inhibit all PNs in a uniform fashion.

### Odor responses at the AL and the MB level of the spiking network model

Figure 1*B* illustrates PN and KC responses to one odor. PNs driven by the stimulus showed a strong transient response at the stimulus onset, a pronounced adaptation during the stimulus, and a period of silence after stimulus offset due to the slow decay of the strong adaptation current. This resembles the typical phasic–tonic response patterns of PNs (Bhandawat et al., 2007; Nawrot, 2012; Meyer et al., 2013).

PNs receiving direct input from ORNs activated by the stimulus showed a strong response at the stimulus onset. Interestingly, the population firing rate over these PNs revealed that the “on” response follows a biphasic profile with an early and a late component. In addition, PNs with no direct input from stimulated ORNs showed an “off” response at the stimulus offset. Nondriven PNs were suppressed during a short period after stimulus onset and showed reduced firing during the tonic response. The PN population response consisted of complex activations of individual PNs with phases of excitation and inhibition. Hence, in the AL, odors were represented as spatiotemporal spike patterns across the PN population.

At the level of the MB, KCs typically show none or very little spiking during spontaneous activity and respond to odors with only a few spikes in a temporally sparse manner (Perez-Orive et al., 2002; Ito et al., 2008; Turner et al., 2008). In our model, synaptic weights between PNs and KCs were tuned to match the very low probability of spontaneous firing. The resulting KC responses were temporally sparse. Because of the negative feedback mediated by strong spike frequency adaptation, most KC spikes were confined to stimulus onset. Notably, we also found that KCs sometimes exhibited off responses. These KC off spikes occurred very rarely, because they are driven by the PN off response, which is much weaker compared with the PN on response. The timing and amplitude of temporally sparse responses are in good quantitative agreement with *in vivo* KC recordings (Ito et al., 2008).

### Dense and dynamic odor representations in the AL

To explore the effects of lateral inhibition and cellular adaptation on stimulus representations, we simulated odor responses in conditions in which we separately deactivated one or both mechanisms. Lateral inhibition was deactivated by setting the inhibitory synaptic weight between LNs and PNs to zero and simultaneously reducing the value of the excitatory synaptic weight between ORNs and PNs, such that the spontaneous firing rate of 8 Hz was kept. Adaptation was deactivated by replacing the dynamic adaptation current by a constant current with an amplitude that maintained the average spontaneous firing rate.

Figure 2 illustrates the separate effects of lateral inhibition and adaptation on odor responses in the PN population. In all conditions, PNs fired spontaneously before stimulation due to spontaneous ORN activation. PNs driven by stimulation receive input from ORNs that were activated by the presented odor. In the absence of adaptation and lateral inhibition (Fig. 2*Ai*,*Bi*), the stimulus response followed the step-like stimulation and showed no further temporal structure. In the presence of lateral inhibition (Fig. 2*Aii*,*Bii*), PNs not driven by the stimulus were strongly suppressed. Adaptation alone (Fig. 2*Aiii*,*Biii*) resulted in a phasic–tonic response profile with a high phasic peak amplitude immediately after stimulus onset. In the presence of both mechanisms (Fig. 2*Aiv*,*Biv*), we observed the characteristic phasic–tonic PN response. The transient response was reduced in peak amplitude, and, interestingly, followed a biphasic profile with an early and a late component.

10.1523/ENEURO.0305-18.2020.f2-1 Figure 2-1 ***A***, ***B***, In the absence of adaptation (***A***, ***B***), lateral inhibition (***B***) sharpens the PN tuning profile (blue). ***C***, ***D***, In the presence of adaptation, the PN tuning profile is not affected by lateral inhibition. The tuning profile was obtained by averaging PN firing rates during the 1 s stimulation window and across 50 trials. PNs receive input from ORNs of the corresponding type according to the receptor response profile. The receptor response profile (gray), rescaled between the minimum and maximum PN firing rates, is shown in all panels for comparison. The insets show the input–output relation between the ORN and the PN firing rates. Both averaged (blue line) and single-trial (gray crosses) PN firing rates are shown. Download Figure 2-1, TIF file.

In our model, the interaction of lateral inhibition and the intrinsic adaptation currents in LNs and PNs account for biphasic PN responses. Because LNs are adapting, lateral inhibition is strongest at stimulus onset. Most PNs were initially suppressed and showed a slightly delayed response, whereas the initial response of PNs with strong input (early component) was not affected. Fast and delayed PN responses have also been found experimentally in the honeybee (Strube-Bloss et al., 2012). Model evidence for the interplay of cellular and network mechanisms behind biphasic PN responses was found in the pheromone system of the moth (Belmabrouk et al., 2011).

### Spike frequency adaptation supports temporal sparseness in the MB

**Figure 3.**
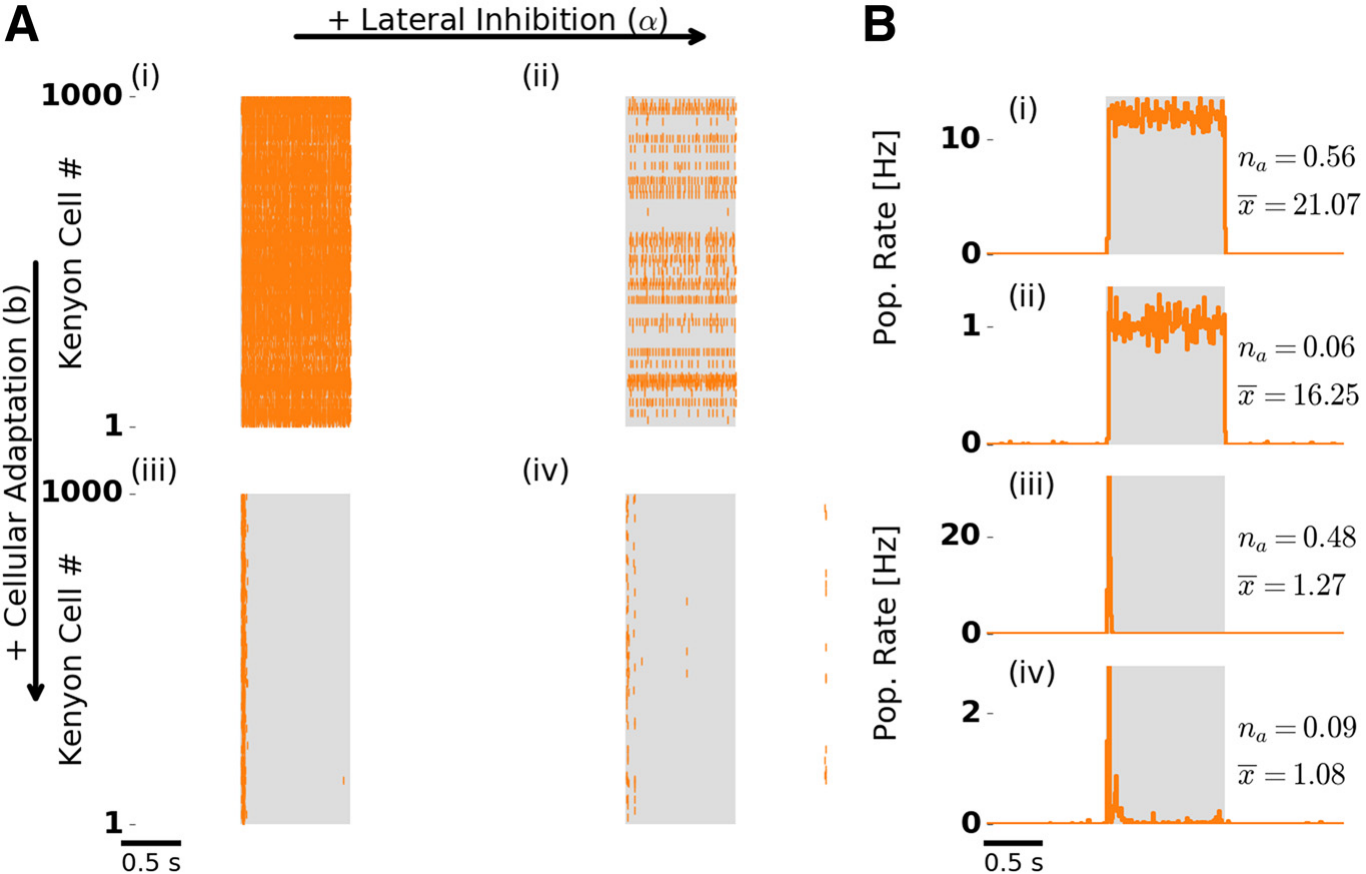
Odor response dynamics of the KC population. Figure layout is as in Figure 2. ***A***, Single-trial population spike raster responses. ***B***, Trial-averaged KC population firing rate. Numbers to the right indicate the fraction of activated KCs (*n_a_*) and the mean number of spikes per activated KC during stimulation ($\bar{x}$). Without adaptation (***Ai***,***ii***, ***Bi***,***ii***), KCs spike throughout stimulation because PN drive is strong and persistent. The fraction of activated KCs drops in the presence of lateral inhibition (***Aii***,***iv***, ***Bii***,***iv***). With adaptation (***Aiii***,***iv***, ***Biii***,***iv***), most of the KC spikes are confined to stimulus onset, indicating temporally sparse responses. We note that spontaneous KC activity is extremely low (0.03 Hz), which is in accordance with previous experimental results (Ito et al., 2008). ***Aiv*** and ***Biv*** are reproduced in Figure 1*B*.

To isolate the contributions of adaptation and lateral inhibition (the latter was present only at the AL level) to odor responses at the MB level, we again tested the four conditions by deactivating one or both mechanisms. In all four conditions, KCs were almost silent and spiked only sporadically during spontaneous activity, whereas the amplitude and temporal profile of their odor response differed across conditions (Fig. 3).

In the presence of adaptation, we observed temporally sparse responses (Fig. 3*Aiii*,*iv*,*Biii*,*iv*). KCs typically responded with only one to three spikes (mean spikes per responding KC were slightly above one; Fig. 3*Biii*,*iv*, compare $\bar{x}$). Because of the negative feedback mediated by strong SFA, most KC spikes were confined to stimulus onset.

In the absence of adaptation and regardless of the presence (Fig. 3*Ai*,*Bi*) or absence (Fig. 3*Aii*,*Bii*) of lateral inhibition, responding KCs fired throughout stimulation, because they received persistently strong input from PNs. Such persistent KC responses are in disagreement with experimental observations (Perez-Orive et al., 2002; Ito et al., 2008; Turner et al., 2008).

**Figure 4.**
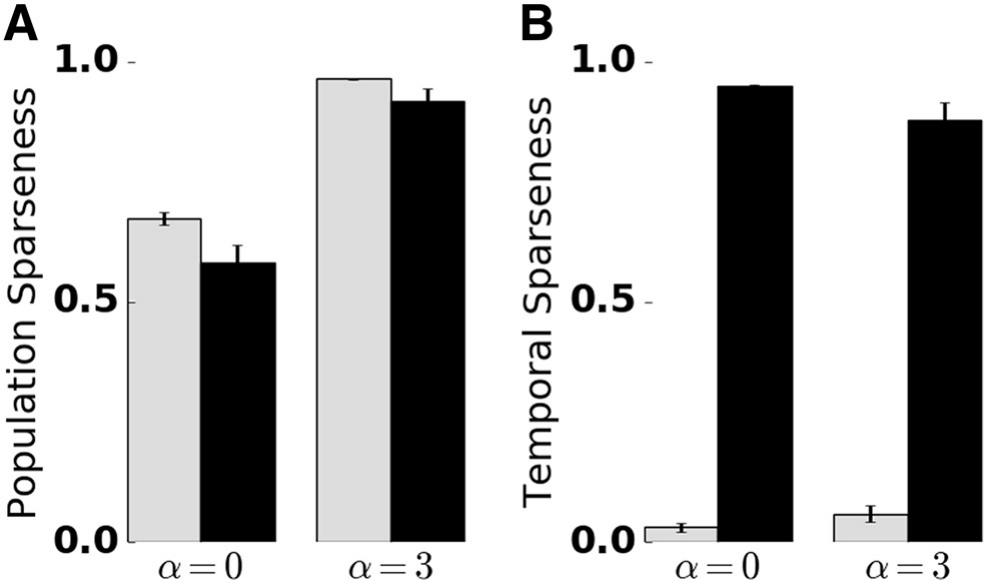
Quantification of temporal and population sparseness in the KC population. Sparseness was measured in the absence (α = 0) and presence (α = 3) of lateral inhibition, and the presence (black bars) and absence (gray bars) of SFA. The sparseness measure was averaged over 50 trials. Error bars indicate SD. A value of 1 corresponds to maximally sparse responses. ***A***, Adaptation promotes temporal sparseness. ***B***, Lateral inhibition in the AL promotes KC population sparseness. Extended Data Figure 4-1 shows temporal sparseness when SFA was disabled in the PN or KC population, and population sparseness for different numbers of PN inputs per KC.

We quantified temporal sparseness of KC responses by calculating a measure modified from (Treves and Rolls, 1991; Materials and Methods). Comparison of temporal sparseness across the four conditions confirms that KC responses were temporally sparse only in the presence of adaptation, whereas lateral inhibition had no effect on temporal sparseness (Fig. 4*A*). Selective absence of adaptation in the KC population (Extended Data Fig. 1-1) did not have an effect on KC temporal sparseness (Extended Data Fig. 4-1*A*). This is due to a high KC spiking threshold that requires strong input and ensures sparse responses. Selective absence of adaptation in the PN population (Extended Data Fig. 1-2) led to persistent tonic KC responses, in addition to the onset KC responses. This is due to strong tonic PN input leading to reduced KC temporal sparseness.

10.1523/ENEURO.0305-18.2020.f4-1 Figure 4-1 ***A***, Temporal sparseness with SFA presence in selected populations. Black: PNs, LNs, and KCs. White dashed: LNs and KCs. White: LNs and PNs. Gray bars indicate simulation in the complete absence of SFA. ***B***, Population sparseness depends on the mean number of PN inputs per KC *k*, both in the absence (*α* = 0, left) and presence (*α* = 3) of lateral inhibition. In comparison with the default number of PN inputs (*k *=* *12, black bars), reducing the mean number of connections to *k *=* *9 (white dashed bars) increased population sparseness, whereas increasing the mean number of connections to *k* = 15 (white bars) decreased population sparseness. The gray bar corresponds to *k *=* *12 in the absence of SFA and is given for reference. Download Figure 4-1, TIF file.

### Lateral inhibition supports population sparseness in the MB

We observed that the fraction of responding KCs was considerably lower in the presence of lateral inhibition (Fig. 3*B*, compare *n_a_* across panels). We recall that lateral inhibition in our model is acting on PNs. The transient PN population rate response showed a biphasic peak in the presence of lateral inhibition. Effectively, the transient PN response was broadened in time and its amplitude was reduced (Fig. 2*B*, compare iii, iv). As a result, KCs received lower peak input from PNs. How does this affect KC responses on a population level?

**Figure 5.**
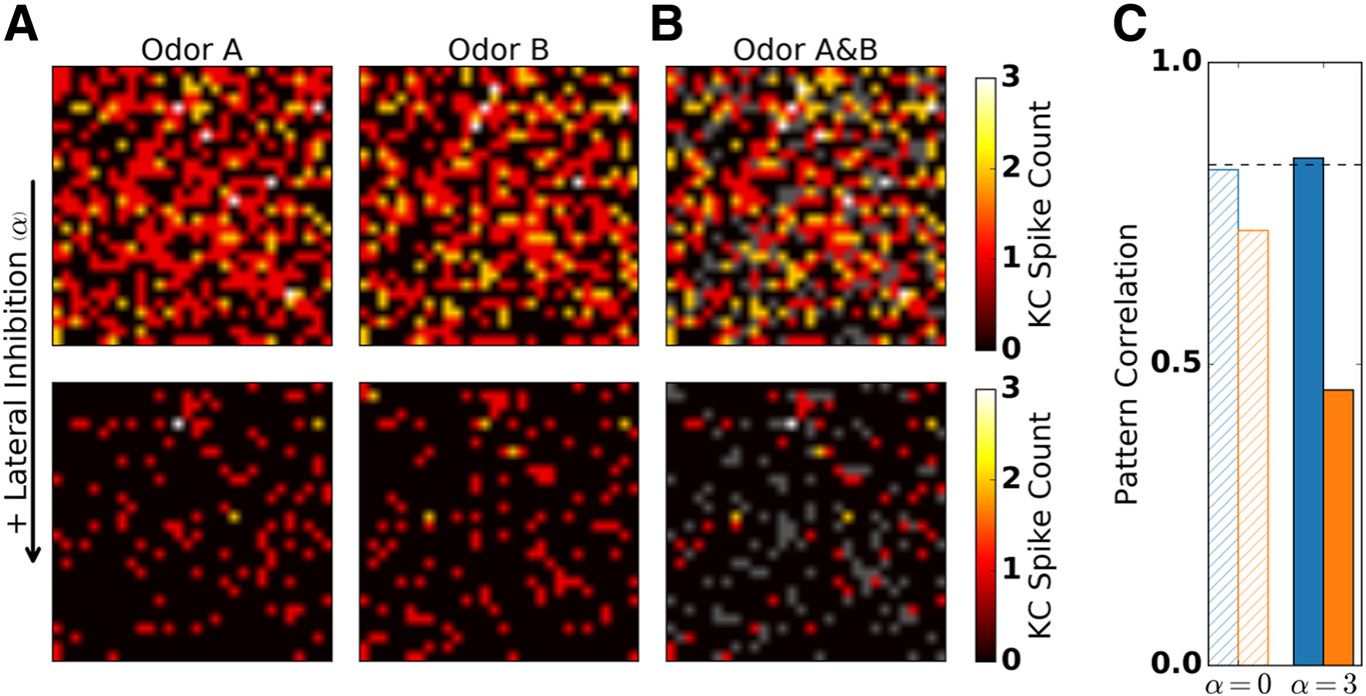
Lateral inhibition in the AL facilitates population sparseness and reduces pattern correlation in the MB. Spike counts (single trial) of 900 randomly selected KCs in response to two similar odors (“Odor A” and “Odor B”) arranged on a 30 × 30 grid in the absence (top row) and in the presence (bottom row) of lateral inhibition. Inactive KCs are shown in black. ***A***, In the absence of lateral inhibition, KCs readily responded to both odors, resulting in an activation pattern where most KCs are active. In the presence of lateral inhibition both odors evoked sparse KC activation patterns. ***B***, Superposition of responses to the two odors. KCs that were activated by both odors are indicated by hot colors (color bar denotes the spike count of the stronger response). KCs that were activated exclusively by one of the two odors are indicated in gray. The fraction of KCs that show overlapping responses is reduced in the presence of lateral inhibition. ***C***, Pattern correlation between the single-trial responses in ***A*** to the two odors obtained for PN (blue) and KC (orange) spikes counts, in the absence (α = 0) and presence (α = 3) of lateral inhibition. Dashed line indicates pattern correlation of the input (ORNs). Pattern correlation was retained at the AL and was reduced at the MB level. Lateral inhibition in the AL reduced pattern correlation in KCs, but not in PNs.

We visualized MB odor representations with activation patterns obtained by arranging KC spike counts evoked by two similar odors on a 30 × 30 grid in arbitrary order (Fig. 5*A*). In the absence of lateral inhibition (Fig. 5*A*, top), a majority of the KC population was activated by both tested odors Each of the 1000 KCs receives input from, on average, 12 PNs and thus from approximately one-third of the total PN population. KCs are readily activated by the strong PN input within a short time window following stimulus onset. Matching experimental results, KCs responded with one to three spikes. Turner et al. (2008) counted 2.2–4.9 KC response spikes in *Drosophila in vivo* intracellular recordings. Using extracellular single unit recordings, Ito et al. (2008) reported that moth KCs typically respond with a single spike and a maximum of five spikes. These numbers correspond to the apparent KC responses in the locust displayed in Broome et al. (2006).

In the presence of lateral inhibition (Fig. 5*A*, bottom), the fraction of activated KCs was reduced substantially (KCs activated; trial averaged, 9%; SD, 3%). Again, this matches well the experimentally reported fraction of stimulus activated KCs in the range of 5–10%, as measured in *Drosophila* (Turner et al., 2008; Honegger et al., 2011) and 6–11% in the locust (Perez-Orive et al., 2002; Broome et al., 2006). In our model, due to the lower peak input from PNs, only KCs with large numbers of PN inputs are likely to be activated. Therefore, the KC population responds more selectively. The range of individual KC responses (one to three spikes) was not affected. These activation patterns demonstrate that the MB odor representations are sparse on a population level, as each odor is represented by the activity of a small fraction of the KC population.

To quantify the population sparseness of odor representations in the MB, we again calculated a sparseness measure (see Materials and Methods). Population sparseness increased in the presence of lateral inhibition, independent of SFA (Fig. 4*B*). In the presence of lateral inhibition and SFA, both population and temporal sparseness were in qualitative and quantitative agreement with experimental findings (Perez-Orive et al., 2002; Szyszka et al., 2005; Ito et al., 2008; Turner et al., 2008). We note that population sparseness also depends on the connectivity parameters of the model (see Discussion). In particular, increasing the average number of PN inputs per KC decreased population sparseness, whereas reducing this number resulted in an increase of population sparseness (Extended Data Fig. 4-1). However, lateral inhibition has a dominant effect on population sparseness, irrespective of the PN–KC connectivity (Extended Data Fig. 4-1). Together, odor representations at the MB level were characterized by a small fraction of the KC population responding with a small number of spikes.

### Decorrelation of odor representations between AL and MB

In our model, lateral inhibition in the AL increased population sparseness of MB odor representations. Does an increased KC population sparseness lead to less overlap between MB odor representations? We visualized the overlap between odor representations in the MB by overlaying KC activation patterns in response to two similar odors (Fig. 5*B*): KCs responding exclusively to odor A or odor B are shown in gray, whereas KCs responding to both odors are color coded. With lateral inhibition (Fig. 5*B*, bottom), most of the KC responses were unique to odor A or odor B, and only a few KCs were activated by both odors. In contrast, with lateral inhibition deactivated (Fig. 5*B*, top), the ratio of KCs with unique responses to the total number of activated cells was low, indicating highly overlapping responses. We quantified the overlap between odor representations evoked by two similar odors in the PN and the KC populations. To this end, we calculated an overlap measure (formally equivalent to Pearson’s correlation coefficient; see Materials and Methods) between spike count patterns evoked by odors A and B (Fig. 5*C*). Interestingly, PNs retained the overlap of the input, independent of lateral inhibition. In contrast, KC representations showed a reduced overlap that decreased even further in the presence of lateral inhibition.

**Figure 6.**
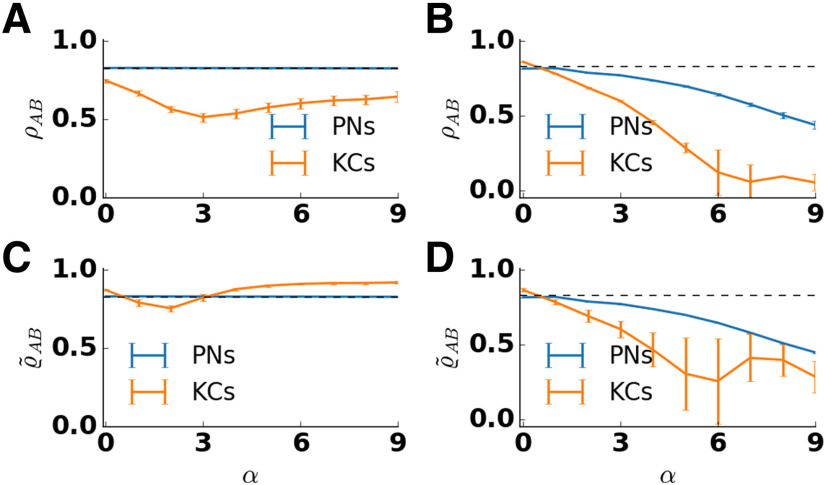
Pattern correlation in the antennal lobe and the mushroom body depend on lateral inhibition strength α. The correlation coefficient ρ_AB_ between the response patterns to two similar odors was calculated and averaged over 50 trials and five network realizations for PNs (blue) and KCs (orange). Error bars indicate SD over trials and network realizations. Pattern correlation of the input is indicated by the dashed line. Input correlation is high because similar odors activate largely overlapping set of receptors. ***A***, In the presence of adaptation, pattern correlation in PNs (blue) stays close to the input correlation for all values of lateral inhibition strength. In KCs (orange), the correlation decreases for small values of lateral inhibition strength and increases for large values of lateral inhibition strength. Pattern correlation in KCs is minimal for α = 3. ***B***, In the absence of adaptation, pattern correlation decreases with the lateral inhibition strength both in PNs and KCs. The decrease is stronger in KCs. ***C***, ***D***, Pattern correlation ${\tilde{\varrho }}_{AB}$ was calculated based on evoked, trial-averaged spike counts in the presence (***C***) and absence (***D***) of lateral inhibition. The correlation coefficient between the trial-averaged response patterns to two similar odors was calculated and averaged over five network realizations. Error bars indicate SD over network realizations. In the presence of adaptation (***C***), the overlap between trial-averaged KC representations of two similar odors (orange) shows a minimum for intermediate strengths of lateral inhibition (1≤α≤3). At the minimum, the KC overlap is below the overlap between trial-averaged PN representations. In the absence of adaptation, the overlap between trial-averaged KC representations is generally lower than the overlap between trial-averaged PN representations for all strengths of lateral inhibition. Extended Data Figure 6-1 and Extended Data Figure 6-2 show the mean fraction of activated KCs and the mean pairwise KC cross-correlation, respectively.

We tested how scaling of the lateral inhibition strength affected the pattern overlap in PN and KC odor representations. To this end, we varied the strength of lateral inhibition (α) in the AL by increasing the strength of inhibitory synapses and adjusting feedforward weights (see Materials and Methods). In addition, we calculated pattern correlations in the absence of adaptation. As before, pattern correlation was calculated for two similar odors that activated an overlapping set of receptors. In the absence of adaptation, lateral inhibition decorrelated odor representations in both populations (Fig. 6*B*). However, for increasing strength of lateral inhibition this leads to an unphysiological regime with an unrealistic low fraction of KCs that show a response (Extended Data Fig. 6-1*B*). In the presence of adaptation, increasing lateral inhibition had different effects on the PN and KC populations (Fig. 6*A*). In PNs, the correlation of the input was retained for all tested values of lateral inhibition. In KCs, pattern correlation first decreased for weak to moderate lateral inhibition strength but then increased for strong lateral inhibition. For an intermediate strength of the inhibitory weights, the pattern correlation between KC responses to similar odors attained a minimal value. For comparison, the bottom panels of Figure 6 show the overlap $\tilde{\varrho }$ between the trial-averaged activation patterns, both in the presence (Fig. 6*C*) and absence (Fig. 6*D*) of adaptation. For PN representations, both measures (ϱ and $\tilde{\varrho }$), indicate the same overlap (Fig. 6*A*,*B* and *C*,*D*, compare blue lines). For KC representations, the measure based on averaged spike counts ($\tilde{\varrho }$) is generally higher, whereas the minimum for intermediate strength of lateral inhibition is shallower (Fig. 6*C*, orange line). Overlap based on spike count patterns recorded in single trials decreases when responses are subject to trial-to-trial variability. In contrast, by averaging the patterns first, the effect of trial-to-trial variability is reduced. The comparison of both overlap measures indicates that in our model KC representations are more variable across trials compared with PN representations.

10.1523/ENEURO.0305-18.2020.f6-1 Figure 6-1 Mean fraction of activated KCs for different strengths of lateral inhibition. We obtained the fraction of activated KCs by counting KCs that have fired at least one spike during one of the given epochs: 1 s of stimulation, 1 s of spontaneous activity, and the first 50 ms after stimulus onset (transient response). ***A***, In the presence of spike frequency adaptation the mean fraction of activated KCs during evoked activity (blue) shows a minimum for the intermediate strength of lateral inhibition. At the minimum, ∼10% of the KCs responded to the stimulus. This fits well with the experimentally reported values in the range of 5–11% (Turner et al., 2008; Honegger et al., 2011). ***B***, In the absence of spike frequency adaptation, the mean fraction of activated KCs decreases with lateral inhibition during evoked activity (blue). Note that for *α* > 4 the fraction of responding KCs is close to zero or is zero. In the absence of spike frequency adaptation, and higher strengths of inhibition, KCs do not receive strong enough inputs to spike. Download Figure 6-1, TIF file.

10.1523/ENEURO.0305-18.2020.f6-2 Figure 6-2 Mean pairwise PN cross-correlation for different strengths of lateral inhibition. For each PN, a vector obtained by binning the corresponding spike train into 50 ms windows was calculated. Pairwise correlation between the vectors was calculated and averaged over all PN pairs and 50 trials. Download Figure 6-2, TIF file.

What is the explanation for the observed minimum in pattern overlap? The minimum of pattern overlap for α = 3 coincides with the minimum of the fraction of activated KCs (Extended Data Fig. 6-1). A lower fraction of responding KCs can be understood as an increased selectivity of KC responses. Both can be linked to changes of the PN input with two counteracting effects. For low strengths of lateral inhibition, the amplitude of transient PN input decreases with lateral inhibition due to temporal dispersion of response spikes across the PN population (Fig. 2*Biv*). KC selectivity increases, whereas pattern overlap decreases.

The increase of pattern overlap for α≥4 is caused by common noise in KCs. The reason for the common noise is the cross-correlation of PN output spike trains. Their mean pairwise cross-correlation is zero in the absence of inhibition and increases with the α value (Extended Data Fig. 6-2). Because of the increased cross-correlation of their inputs, KCs are more easily activated. However, for α≥4, KC responses are increasingly stimulus unspecific due to common noise and overlapping inputs. Together, for weak to intermediate lateral inhibition KC selectivity increases, responses remain stimulus specific and become more sparse. For strong lateral inhibition (α≥4), the fraction of activated KCs increases as KC responses become more unspecific, driven by common noise.

In general, a reduction of pattern correlation from PN to KC representations is characteristic for the insect MB (Laurent, 2002). Furthermore, low overlap between KC representations has been found to facilitate the discrimination of odors (Campbell et al., 2013). We therefore choose the intermediate strength of the inhibitory weights (α = 3) as a reference point in our model.

### Odor encoding on short and long timescales

**Figure 7.**
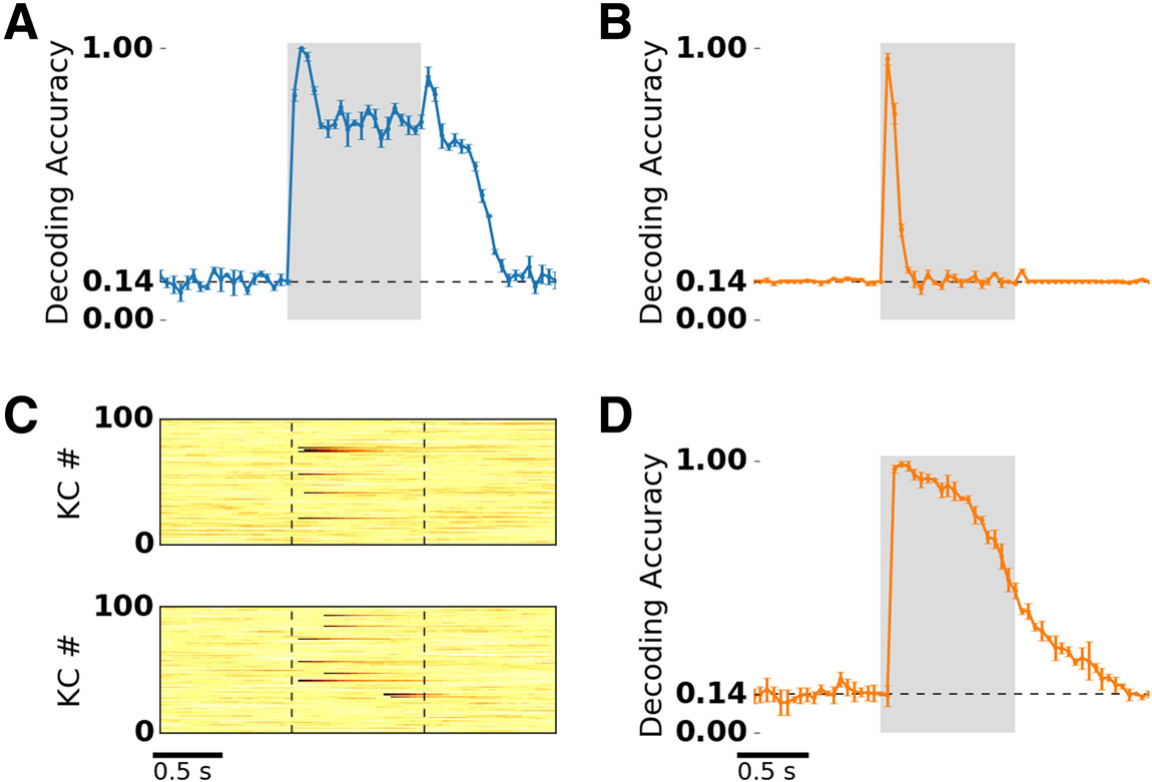
Decoding of odor identity indicates a prolonged and reliable odor information in KC adaptation currents. ***A***, ***B***, ***D***, Decoding accuracy was calculated for nonoverlapping 50 ms time bins, based on a set of seven stimuli (chance level, ∼0.14) presented for 1 s (shaded area). Blue shading indicates SD obtained from a cross-validation procedure (see Materials and Methods). ***A***, Decoding of odor identity from PN spike counts. Decoding accuracy peaks at odor onset and offset, and remains high after stimulation. ***B***, Decoding of odor identity from KC spike counts. Decoding accuracy is above chance only in the first three bins following stimulus onset. ***C***, Adaptation current amplitudes (single trial, hot colors in arbitrary units) of 100 selected KCs in response to “odor A” (top) and “odor B” (bottom). ***D***, Decoding of odor identity from KC adaptation currents. Decoding accuracy peaks 150 ms after odor onset then drops during stimulation, but remains high and is sustained after odor offset.

Next, we tested whether in our model the information about stimulus identity is contained in AL and MB odor representations by performing a decoding analysis in subsequent time bins of 50 ms (see Materials and Methods). In PNs, decoding accuracy peaked during stimulus onset and offset (Fig. 7*A*). Both peaks coincide with a state of transient network activity caused by the odor onset or offset. The on and the off responsive PNs establish odor representations optimized for discrimination. After stimulus onset, decoding accuracy dropped but remained on a plateau well above chance level. Remarkably, after stimulus offset, odor identity could be decoded for an extended time period (several hundreds of milliseconds), albeit with a reduced accuracy. Such odor aftereffects have been demonstrated previously in experiments (Szyszka et al., 2011; see Discussion).

In KCs, decoding accuracy was above chance level only in the first two to three time bins (∼100 ms) after stimulus onset (Fig. 7*B*). In all other time bins, decoding accuracy remained at chance level. Because the spiking activity in the KC population is temporally sparse, the continuous information at the AL output is lost in the MB spike count representation. This raises the question of whether and if so, then how the information could be preserved in the MB throughout the stimulus. The intrinsic timescale of the adaptation currents might potentially support prolonged odor representations (Fig. 7*C*). We therefore repeated the decoding analysis on the adaptation currents measured in KCs (Fig. 7*D*). Indeed, the stimulus identity could reliably be decoded based on the intensity of the adaptation currents in subsequent time bins of 50 ms. Decoding accuracy peaked after stimulus onset and then slowly decreased. Remarkably, the timescale of the decay is comparable during and after stimulation. Because KCs show very little spontaneous activity, the decoding accuracy after stimulation decays with the adaptation time constant. This is due to the exponential decay of the adaptation currents evoked by stimulation and the stochastic adaptation current fluctuations in the background due to channel noise.

## Discussion

We investigated the transformation between dense AL and sparse MB odor representations in a spiking network model of the insect olfactory system. Our generic model demonstrates lateral inhibition and spike frequency adaptation as sufficient mechanisms underlying dynamic and combinatorial responses in the AL that are transformed into sparse MB representations. To simulate responses to different odors, we incorporated simple ORN tuning and glomerular structure in our model. This approach allows us to investigate how different odors are represented in the AL and MB population activity and to assess information about odor identity contained in respective odor representations. We inspected overlap between odor representations in both populations. Sparse coding reduces overlap between representations, as has been predicted on theoretical grounds (Marr, 1969; Albus, 1971; Kanerva, 1988) and has been shown for MB odor representations (Szyszka et al., 2005; Turner et al., 2008; Lin et al., 2014). Similarly, our model shows pattern decorrelation in the MB but not in the AL.

### Postodor responses

In our model, we found on and off responsive PNs. At the stimulus offset, the off responsive PNs transiently increase, whereas the on responsive PNs transiently decrease their firing rate (Fig. 2). On responsive PNs remain adapted beyond stimulus offset. Their excitability thus stays reduced until the slow adaptation current has decayed. In contrast, in off responsive PNs increased lateral inhibition during stimulation causes a below-baseline adaptation level throughout the stimulus and thus an increased excitability. In effect, the odor-evoked and the postodor PN activation patterns are reversed (i.e., anti-correlated; data not shown). This result matches well the experimental observations in the honeybee (Szyszka et al., 2011; Nawrot, 2012; Stierle et al., 2013) and *Drosophila* (Galili et al., 2011) PNs. Those results show highly correlated response patterns throughout stimulation and stable, but anti-correlated, postodor response patterns.

### Differential mechanism underlying temporal and population sparseness in KCs

In our model, the two mechanisms underlying temporal sparseness and population sparseness act independently.

Temporal sparseness of KC responses in our model compares well to the experimentally recorded spiking responses in *Drosophila*, locust, and moth (Perez-Orive et al., 2002; Ito et al., 2008; Turner et al., 2008), and to calcium imaging experiments in the honeybee (Szyszka et al., 2005). The model proposed here solely relies on spike frequency adaptation for temporally sparse responses. On a cellular level, strong adaptation currents in KCs, which are suitable for the generation of sparse responses, have been found in the honeybee (Wüstenberg et al., 2004) and cockroach (Demmer and Kloppenburg, 2009). In the model, temporal sparseness is not affected by the deactivation of lateral inhibition, a finding supported by a previous study by Farkhooi et al. (2013).

Several studies have suggested that either feedforward inhibition (Assisi et al., 2007) or feedback inhibition (Szyszka et al., 2005; Papadopoulou et al., 2011; Gupta and Stopfer, 2012; Lei et al., 2013; Kee et al., 2015) causes temporally sparse responses. The existence of inhibitory feedback neurons in the MB has been demonstrated experimentally in different insect species (cockroach: Takahashi et al., 2017; *Drosophila*: Liu and Davis, 2009; honeybee: Grünewald, 1999; locust: Papadopoulou et al., 2011), whereas evidence for feedforward inhibition to the MB is lacking (Gupta and Stopfer, 2012). Our model demonstrates that temporally sparse responses can be obtained without an inhibitory circuit motive. There is further evidence for a GABA-independent mechanism for the temporal shortening of KC responses. Calcium imaging studies in *Drosophila* (Lei et al., 2013; Lin et al., 2014) and in the honeybee (Farkhooi et al., 2013; Froese et al., 2014) showed that the temporal profile of the fast response dynamics of KCs is preserved even if GABAergic inhibition is blocked.

What could be the benefit of temporally sparse responses in KCs? We hypothesize that temporal sparseness is an important strategy for the system to follow fast transient inputs rather than representing static input. The typical laboratory experiment uses controlled odor stimuli that are presented with static intensity for up to several seconds. However, in a natural setting, olfactory inputs are highly dynamic (Vickers et al., 2001). Natural odor plumes do not represent a gradient intensity due to diffusion. Rather, odors distribute in space and time in a filamentous structure (Vickers, 2000; Celani et al., 2014), and filaments from different odors do not mix perfectly (Szyszka et al., 2012). Because of wind and animal movement, particularly relevant for flying insects, the olfactory input will generally be highly dynamic in time, resulting in fast and steep changes of odor concentration whenever the animal encounters an odor filament. In such an on–off scenario, temporally sparse responses in KCs might enable the processing of rapid odor filament encounters. We hypothesize that the KC population provides a temporally sparse representation of the odor identity of each filament with a single spike or a few spikes in each KC. The system is thus able to track individual odor filament encounters over time, and the animal can adapt its behavior accordingly (e.g., during odor source location in foraging flights; Budick, 2006; Van Breugel and Dickinson, 2014; Egea-Weiss et al., 2018). At the periphery, it has been shown that the olfactory receptor neurons in various insect species are able to follow fast repeating olfactory input pulses even for high pulse frequencies (Vickers et al., 2001; Szyszka et al., 2014). Our results show that the mechanism of spike frequency adaptation is able to generate temporally sparse responses to the onset of an odor and thus to detect temporal changes in the olfactory input rather than encoding the persistence of a stimulus. Adaptation has previously been implicated as a means to compute the temporal derivative of sensory input (Lundstrom et al., 2008; Tripp and Eliasmith, 2010; Farkhooi et al., 2013). A second advantageous property of spike frequency adaptation is that it facilitates the reliability of individual responses and significantly reduces the variability in the number of response spikes across repeated stimulus representation (Farkhooi et al., 2011, 2013). Temporal sparseness is not limited to the insect MB and has been discovered in diverse sensory systems, notably in mammalian sensory cortices (Vinje and Gallant, 2000; Hromádka et al., 2008; Isaacson, 2010; Wolfe et al., 2010), where it has also been linked to the encoding of temporally dynamic input in natural sciences (Haider et al., 2010; Yen et al., 2010). We suggest that SFA is a general mechanism across sensory systems and taxa supporting reliable temporally sparse responses under natural sensory input conditions.

The KC population sparseness in our model matches qualitatively and quantitatively with experimental estimates from electrophysiological responses in locust and *Drosophila* (Perez-Orive et al., 2002; Turner et al., 2008) and from calcium imaging in *Drosophila* (Honegger et al., 2011). Our model shows sparse KC responses on a population level in the presence of, but not in the absence of, lateral inhibition. Calcium imaging experiments in the honeybee (Froese et al., 2014) have shown that inactivating GABA transmission disrupts population sparseness, which is in line with our modeling results. In *Drosophila*, feedback inhibition contributes to the population sparseness of KCs, as blocking of feedback inhibition reduced population sparseness and undermined the learned discrimination of similar odors (Lei et al., 2013; Lin et al., 2014). In addition, a cellular mechanism such as a high threshold for KC activation in *Drosophila* (Turner et al., 2008) and active KC subthreshold properties in locust (Perez-Orive et al., 2002; Jortner et al., 2007) have been shown to support population sparseness. Moreover, the plasticity of inhibitory feedback changing response patterns in the KC population might be crucial for associative learning (Liu and Davis, 2009; Haehnel and Menzel, 2010; Filla and Menzel, 2015; Haenicke et al., 2018). We suggest that different neurophysiological mechanisms of sparseness are not mutually exclusive but rather act in concert. Both lateral inhibition in the AL and feedback inhibition in the MB are likely to be necessary for sparse KC population responses.

Evidently, the sparse connectivity scheme between the PN and KC populations is the anatomic basis for population sparse response patterns in the KC layer (Nowotny et al., 2005; Jortner et al., 2007; Huerta and Nowotny, 2009). This connectivity is divergent-convergent with an apparent high degree of randomness (Caron et al., 2013). In our model, connectivity is parametrized by the average number of inputs *k* per KC and by the synaptic weight of PN-KC (*w*_PK_). Experimental estimates indicate a small number of inputs per KC. Anatomical data in *Drosophila* provided estimates of *k* ∼ 13 (Turner et al., 2008) and k≈5−7 (Leiss et al., 2009). Szyszka et al. (2005) estimated *k ∼* 10 inputs per KC for the honeybee. For our model, we chose *k* = 12. Increasing or decreasing this number resulted in a decrease or increase of population sparseness, respectively (Extended Data Fig. 4-1). Importantly, with respect to population sparseness, the physiological mechanism of lateral inhibition and anatomic connectivity parameters represent conceptually distinct factors. Neuromodulation can affect lateral inhibition on short (tens to hundreds of milliseconds) timescales (Lizbinski and Dacks, 2018). Our results indicate that this modulation could have a drastic effect on population sparseness in the MB. The number of connections, in contrast, can be considered stable on short timescales. However, on a long timescale (days) experience-dependent structural plasticity has been demonstrated within the synaptic densities of *Drosophila* MB calyx, where KCs connect to presynpatic PN boutons (Kremer et al., 2010).

### Decorrelation of odor representations between AL and MB

Decorrelation of stimulus representations has been postulated to be a fundamental principle underlying sensory processing (Barlow, 1961, 2001). In particular, in the olfactory system odor representations are transformed to decorrelate activity patterns evoked by similar odors, making them more distinct (Uchida et al., 2013; Friedrich and Wiechert, 2014; Galizia, 2014). Transformations decreasing the overlap between representations are termed pattern decorrelation. Less overlapping representations increase memory capacity (Treves and Rolls, 1991) and make the discrimination of odors easier (Campbell et al., 2013). In our model, we found that AL odor representations preserved the similarity of the input, whereas representations of similar odors at the periphery became decorrelated in the MB.

We quantified the effects of lateral inhibition and adaptation on pattern correlations. We found that decorrelation of activity patterns in the AL occurred only in the absence of adaptation. Moreover, the amount of decorrelation depended on lateral inhibition strength. Considering decorrelation of odor representations, the difference between lateral inhibition and adaptation is substantial. In our model, lateral inhibition alone sharpens PN responses, whereas adaptation leads to linearization of the input–output relation between the input from ORNs and the PN output (Extended Data Fig. 2-1). In computational studies, lateral inhibition was previously shown to decorrelate odor representations (Luo et al., 2010; Schmuker et al., 2011). In a *Drosophila* study using single sensillum recordings from ORNs and whole-cell recordings from PNs, lateral connection in the AL were found not to affect correlations between glomerular channels (Bhandawat et al., 2007), but there is also evidence for decorrelation of AL representations (Olsen et al., 2010). In our model, pattern correlation between representations of similar odors was preserved at the level of the AL but was reduced in the MB. The observed counteracting effect of adaptation on pattern decorrelation by lateral inhibition in the AL is generally valid for strong adaptation. Strong adaptation currents provide slow, negative feedback that has a linearizing effect on the input–output relation (Ermentrout, 1998). As a consequence of strongly adapting PNs in our model, the pattern correlation of AL odor representations is equal to the pattern correlation given by the tuning profile of the ORN input (Fig. 6).

### Odor representation in adaptation currents

Early investigations of dynamical odor representations have shown that odor identity can be reliably decoded from PN spike counts in 50 ms time bins (Stopfer et al., 2003; Mazor and Laurent, 2005; Krofczik et al., 2009). We used this approach to show that odor representations were specific and reliable in our model, including both AL and MB odor representations. We found that odor representations were optimized for discrimination during odor onset (Fig. 7*B*,*C*). Optimal decoding during stimulus onset is in agreement with electrophysiological evidence from locust and honeybee PNs (Mazor and Laurent, 2005; Krofczik et al., 2009). In the auditory system, Hildebrandt et al. (2015) found that grasshoppers use the onset of a sound pattern as the most reliable information for sound localization. Their study provides behavioral evidence that, in the presence of adaptation, the onset response preserves absolute stimulus levels. Our model shows that at the MB level, stimulus identity could be decoded from KC spike counts only during a short time window after stimulus onset (up to ∼150 ms; Fig. 7*B*). This is a consequence of the temporally sparse KC responses.

Moreover, we found that KC adaptation currents retain a representation of stimulus identity, resembling a prolonged odor trace (Perisse and Waddell, 2011; Dylla et al., 2013). In our model, an odor trace present in adaptation levels extends well beyond the brief spiking responses. Adaptation currents constitute an internal dynamical state of the olfactory network that is not directly accessible to downstream neurons: a “hidden state” (Buonomano and Maass, 2009). However, adaptation levels influence the responses to (subsequent) stimuli (Farkhooi et al., 2013) and may also affect downstream processing through an indirect pathway.

Our results suggest that odor representations are not exclusively found in the spiking activity. The phenomenological model of spike-triggered adaptation used in this study (see Materials and Methods; for review, see Benda and Herz, 2003) is motivated by calcium-activated outward potassium currents. Those currents are activated by spike-triggered calcium influx, which is only slowly removed. We propose that information carried by temporally sparse KC spikes is stored on prolonged timescales by the slowly decaying calcium concentration. We predict long-lasting levels of calcium in the KC population that retain odor information and provide a potential substrate for a short-term sensory memory. Therefore, classification of calcium levels recorded in the MB should reveal odor identity on a timescale determined by the decay of the intracellular calcium level. Indeed, a recent study by Lüdke et al. (2018) showed that prolonged calcium activity in KCs encoded odor information and could be related to behavioral odor recognition performance in trace conditioning experiments where a conditioned odor stimulus is followed by a temporally delayed reinforcement stimulus.

## Synthesis

Reviewing Editor: Tatyana Sharpee, The Salk Institute for Biological Studies Decisions are customarily a result of the Reviewing Editor and the peer reviewers coming together and discussing their recommendations until a consensus is reached. When revisions are invited, a fact-based synthesis statement explaining their decision and outlining what is needed to prepare a revision will be listed below. The following reviewer(s) agreed to reveal their identity: Jan Benda, Tatyana Sharpee.

This manuscript analyzes the impact of lateral inhibition and spike frequency adaption on the sparseness of responses in the olfactory system. The authors show that spike frequency adaptation increases the sparseness of responses in time whereas lateral inhibition contributes to sparseness at the population level. However, the lateral inhibition impacts sparsenee in a non-monotonic manner in the presence of spike frequency adaptation. There is a certain value of lateral inhibition that leads of maximal sparseness of Kenyon cell responses, and that either weaker or stronger lateral inhibition leads to less sparse stimuli. It is important to investigate the model and provide a mechanistic insight for why this non-monotonic effect takes place. The detailed comments from two reviewers follow.

Reviewer 1:

In their manuscript the authors use simulations based on leaky-integrate-and-fire neurons to compare the effects of spike-frequencyadaptation and lateral inhibition on the temporal and population sparseness in the insect olfactory system. The simulations are done on a well chosen abstraction level and reproduce experimental firing properties of PN, LN, and KC cells sufficiently well. The conclusions are valid, although not surprising - spike-frequency adaptation is responsible for temporal sparseness and lateral inhibition for population sparseness. Unfortunately the manuscript mainly restricts itself to describe the simulation results without attempting to explain some of the not so obvious findings.

The paper could be considerably improved by coming up with mechanistical explanations of some of the findings, and by better disecting the role of spike-frequency adaptation as I explain below. Doing so would add tremendously to our understanding of the generation of sparse codes.

Major issues

------------

Context Fig 5: How can this be understood given the PN responses? Ho does this depend on PN-KC connection parameters?

Context Fig 6 (Results and Discussion): This is where things get interesting! Can you explain the minimum of pattern correlation in Fig 6A mechanistically?

If the aim of the system is to generate as distinct patterns as possible then why should it have spike frequency adaptation at all? Why is it advantagous to have temporal sparseness and thus spike-frequency adaptation at all? Why and where is there a trade-off between temporal and population sparseness, and thus between adaptation and lateral inhibition?

Spike-frequency adaptation (SFA), also discussion line 397-405: you have SFA in all three cells (PN, LN, AND KC) and as I understand it correctly you turn it off in all three cell types. Can you dissect this? What happens if you have SFA in only one of the cell types? Will you also get temporal sparseness? Also consider Hildebrandt, Ronacher, Hennig, Benda (2015) PLOS Biology “A Neural Mechanism for Time-Window Separation Resolves Ambiguity of Adaptive Coding” when discussing the role of SFA in KC cells!

Minor issues

------------

page 4, receptor input: are real receptor responses not adapting and really Poisson? I am not an expert for olfactory systems, but in any other system receptor responses do adapt (to about 50 % of the initial peak firing rate) and strong responses are definitely not Poisson like. Please motivate your choice.

Eq 10, lines 197-200: What is your rational to put noise only into the adaptation current? This also implies that in your scenarious without adaptation, there is no noise source at all in the system. I would instead add whhite noise to the current equations 2 - 4.

Tab 2, Eq 2-4: All three neuron types are modelled with the same parameter (membrane time constants, leak, threshold, etc). Can you cite some literature to motivate this?

page 7 simulation conditions: for the “no adaptation” scenario you replace the adaptation current by a constant one to ensure the right firing regime. For the “no lateral inhibition” scenario you scale synaptic weights instead. In this way you also modify temporal dynamics of input currents, in particular because tau_E < tau_I. Why not also just putting in compensatory currents?

Fig 1 A: Add arrows to the lateral inhibition

Fig 2 B also indicate that x-axis is time

Fig 2: Add panels showing firing rate averaged over some time interval

as a function of PN neuron # and discuss these in the text.

Fig 4 B: alpha=0 population sparseness: the values should depend on some of your model parameter (in particular PN-KC connections). Can you elaborate on this?

Line 307 & 421: Can you specify what is quantitatively similar to the experimental data? Mean firing rates? Adaptation time constants? etc.

Fig 5 A bottom & line 316-317: I can not really follow your statements given this example: From the 5 white dots in the A&B case 4 are from B and only one from A, whereas two from A seem to be shared by B.

Line 366: no it is no tthe adaptation noise it is simply the adaptation current that is increased by the spike but that decays with a slow time constatn and in this way carries information about previous spikes into the future.

Discussion lines 423-435 and 446-454: how exactly do all these experimental findings relate to your model. Is forward inhibition different from SFA? In what ways? Are your conclusion really very general or are there other possibilities that would change the picture? Please really discuss these issues.

Discussion line 474ff: simply explain that the calcium flows in because of the (temporal sparse) spikes. Becasue calcium is removed from the intracellular space only with a slow time constant the information of the spikes is still there after quite a while.

Reviewer 2:

This manuscript is well written and systematically analyzes the contribution of spike frequency adaptation and lateral inhibition to the temporal and population sparseness of population responses in the olfactory system.

There a number of minor comments that need to be addressed:

Line 100, not clear sentence that “simulations were initialized for 2000 ms without recording the activity” Please clarify how the simulations were initialized.

In Eq. 5 please clarify how spikes were generated, was it using Poisson process with rate from Eq. 1?

Lines 313-314, the sentence that starts “Given sparse population responses” needs to be rephrased.

Lines 345, the information about the stimulus identity has to be present in AL and MB representation. Maybe rephrase to state such as “The data were analyzed to determine the time course with which stimulus identity could be decoded from AL and MB responses”

Lines 387, double period.

## Author Response

Synthesis of Reviews:

Computational Neuroscience Model Code Accessibility Comments for Author (Required):

Code is provided

Significance Statement Comments for Author (Required):

I would suggest to edit 1st, 2nd, and 5th sentences.

Some of the suggested changes:

1) “In trace conditioning insects” --> “The trace conditioning insects”

We have changed this sentence as follows:

“In trace conditioning insects, like vertebrates, are able to ...” -> “In trace conditioning experiments, insects, like vertebrates, are able to ...”

2) “To form the association” --> “To form this association”

Done

3) We found that odor identity can be encoded ...

Done

In addition it might be worthwhile to mention the non-monotonic dependence of sparseness on lateral inhibition.

We have now added this result to the abstract rephrasing the second-to-last paragraph as follows:

“The interplay of both mechanisms shapes spatio-temporal odor representations, which are optimized for discrimination of odors during stimulus onset and offset. Response pattern correlation across different stimuli showed a non-monotonic dependence on the strength of lateral inhibition with an optimum at intermediate levels, which is explained by two counter-acting mechanisms.”

We did not mention it again in the significance statement, which we deliberately kept simple and straight, pointing to the mechanism of SFA and it’s implication of an intracellular substrate for the so-called “odor-trace”.

Comments on the Visual Abstract for Author (Required):

n/a

We have now generated a Visual Abstract that will be uploaded with the revised MS and Figure files.

Synthesis Statement for Author (Required):

This manuscript analyzes the impact of lateral inhibition and spike frequency adaption on the sparseness of responses in the olfactory system. The authors show that spike frequency adaptation increases the sparseness of responses in time whereas lateral inhibition contributes to sparseness at the population level. However, the lateral inhibition impacts sparsenee in a non-monotonic manner in the presence of spike frequency adaptation. There is a certain value of lateral inhibition that leads of maximal sparseness of Kenyon cell responses, and that either weaker or stronger lateral inhibition leads to less sparse stimuli. It is important to investigate the model and provide a mechanistic insight for why this non-monotonic effect takes place. The detailed comments from two reviewers follow.

We thank the editor for her constructive summary of the reviews. We fully agree that the manuscript will benefit from a mechanistic analysis of the model. As outlined below we have now included additional detailed analyses, have extended Figure 6 and added six new Supporting Figures.

Reviewer 1:

In their manuscript the authors use simulations based on leaky-integrate-and-fire neurons to compare the effects of spike-frequency adaptation and lateral inhibition on the temporal and population sparseness in the insect olfactory system. The simulations are done on a well chosen abstraction level and reproduce experimental firing properties of PN, LN, and KC cells sufficiently well. The conclusions are valid, although not surprising - spike-frequency adaptation is responsible for temporal sparseness and lateral inhibition for population sparseness. Unfortunately the manuscript mainly restricts itself to describe the simulation results without attempting to explain some of the not so obvious findings.

The paper could be considerably improved by coming up with mechanistical explanations of some of the findings, and by better disecting the role of spike-frequency adaptation as I explain below. Doing so would add tremendously to our understanding of the generation of sparse codes.

We thank the reviewer for her/his positive evaluation of our work. We address her/his constructive request for mechanistic explanation in additional model analyses as outlined in our point 2 point reply below. We are presenting new results in additional figure panels and supporting figures, and we have explained the mechanistic analyses in the Results and Discussion section of the revised manuscript text, accordingly.

Major issues

------------

#1 Context Fig 5: How can this be understood given the PN responses? Ho does this depend on PN-KC connection parameters?

We have now thoroughly revised the results section “Lateral inhibition supports population sparseness in the MB” on pages 16/17 to better explain how lateral inhibition affects population sparseness in Fig. 5AB.

Evidently, KC population sparseness also depends on the PN->KC connectivity. In our model this is parameterized by the mean number k of PN inputs per KC and a (fix) synaptic weight. Each of the 1.000 KCs receives input from, on average, 12 PNs (this being in the range of experimental estimates between 7 and 20), i.e. from about 1/3rd of the total PN population. Thus, a large fraction of the PNs are activated by any given odor stimulus and consequently KCs with overlapping inputs are most likely to be activated.

In the new additional analysis of supporting Figure 4-1B we have now analyzed population sparseness for values of k = 9,12,15 in the presence (alpha = 3) and absence (alpha = 0) of lateral inhibition. As expected a lower indegree (k=9) increases sparseness in the absence of lateral inhibition by almost 30%, while a higher k=15 decreases sparseness by more than 30%. However, in all cases, including lateral inhibition clearly shows the far bigger effect on sparseness resulting in values of S >0.9 for k=9,12 and doubling sparseness for k=15. These additional results and the supporting figure 4-1 are now mentioned in the results lines 358 ff.

Importantly, with respect to population sparseness, connectivity and lateral inhibition represent conceptional distinct factors. Lateral inhibition can be modulated physiologically on short time scales (tens to hundreds of ms). While in the current manuscript we make not use of neuromodulation of lateral inhibition, this might become relevant in future model studies. The number of connections and connection strength, however, are comparably stable. We have now added a paragraph in the Discussion (lines 561 ff) to relate to the difference of structural parameters and lateral inhibition and to compare our choice for k with experimental estimates of k.

#2 Context Fig 6 (Results and Discussion): This is where things get interesting! Can you explain the minimum of pattern correlation in Fig 6A mechanistically?

If the aim of the system is to generate as distinct patterns as possible then why should it have spike frequency adaptation at all? Why is it advantageous to have temporal sparseness and thus spike-frequency adaptation at all? Why and where is there a trade-off between temporal and population sparseness, and thus between adaptation and lateral inhibition?

We agree that this is an interesting aspect and we have improved the manuscript accordingly.

The origin of the minimum of pattern correlation is due to two opposing effects and in the revised MS we now provide a detailed mechanistic explanation on pages 18/19. This explanation is supported by an improved analysis of pattern correlation in dependence on the strength of lateral inhibition (cf. Methods, additional panels Fig. 6 C,D) and through Supporting Figures. 6-1 and 6-2.

Our model shows that minimizing the pattern overlap alone is not a priority of olfactory processing. In fact, our model shows that in the presence of SFA, MB odor representations can be successfully decoded even for similar odors, in a regime with intermediate strength of lateral inhibition, sparse, biologically realistic responses and reduced overlap between KC representations. In the absence of SFA overlap can be reduced more with stronger lateral inhibition, but this leads to an unphysiological regime with unrealistic low numbers of KCs that show a response (supporting Fig. 6-1B). These responses are strong and continuous in contrast to the experimentally observed temporally sparse response with only very few spikes. Our model proposes that a trade off can be avoided by an operating regime with SFA and lateral inhibition that allows for efficient sparse encoding.

Why temporal sparseness? Temporal sparseness effectively limits the olfactory systems’ spiking response at the level of KCs to an onset response following a step-like input. Under natural conditions, the olfactory scene is a complex composition of various odors from different sources. Odors distribute in space and time in a filamentous structure and filaments from different odors do not mix perfectly. Due to wind and animal movement - particularly relevant for flying insects - the olfactory input can be highly dynamic in time. This results in fast and steep changes of odor concentration when encountering odor filaments similar to step-like increases. Due to animal movement through filamentous odor plumes the system encounters highly fluctuating input as in an on-off-on scenario. The hypothesis is that temporal sparseness might be an important strategy for the system to follow fast transient inputs rather than representing static input. Temporal sparseness has also been established in mammalian sensory cortices where it has also been linked to the encoding of temporally dynamic input. We have now added a discussion paragraph on the possible role of temporally sparse odor encoding at the level of KCs and on the role of spike frequency adaptation in generating temporal sparseness in lines 510.

Why SFA in our model? One important reason is the fact that strong adaptation currents have been described in KCs experimentally (Demmer & Kloppenburg, 2009). The second important reason is - and this is one of the major messages of this manuscript - that SFA can account for the experimentally demonstrated temporal response sparseness. This hypothesis deviates from the previously suggested inhibitory circuit motives. SFA is a robust mechanism because it is a cellular property and thus distributed across the system and because it does not require adjustment of inhibitory time constants in a feedback circuit. Moreover, SFA has been implicated as a mechanism for computing a (partial) time-derivative and thus for focusing responses on temporal input changes rather than on static input. We believe this might be a general mechanism across sensory systems and across taxa. What’s more, it has been shown that SFA reduces trial-by-trial variability of spike responses to repeated stimulation. We have revised the discussion accordingly.

In effect, and similar to the results in Hildebrandt et al., 2015, spiking responses in KCs follow steep transients and have a normalizing effect on the number of response spikes. The short responses lead to a fast diminishing of odor representation in the spiking responses; however, we propose that SFA currents in the KC population retain what has been termed the “odor trace” and thus compensate for the extinct memory in the population spiking activity. We have here only tested decoding of odor identities and not intensities. This would have required a sophisticated model for transduction and transformation in receptor neurons which is beyond the scope of the present MS. However, in ongoing work we further develop our model to incorporate a versatile receptor model incorporating very recent experimental findings in Drosophila melanogaster.

#3 Spike-frequency adaptation (SFA), also discussion line 397-405: you have SFA in all three cells (PN, LN, AND KC) and as I understand it correctly you turn it off in all three cell types. Can you dissect this? What happens if you have SFA in only one of the cell types? Will you also get temporal sparseness? Also consider Hildebrandt, Ronacher, Hennig, Benda (2015) PLOS Biology “A Neural Mechanism for Time-Window Separation Resolves Ambiguity of Adaptive Coding” when discussing the role of SFA in KC cells!

To dissect the effects of the presence of spike-frequency adaptation (SFA) in different cell types, we have rerun simulations with SFA selectively disabled in one cell type. We have added two supporting figures showing the odor response with SFA selectively disabled in KCs (Fig. 1-1), and with SFA selectively disabled in PNs (Fig. 1-2). In the absence of SFA in KCs, we have observed temporally sparse responses, albeit with reduces sharpness of the responses (cf. Fig 4-1A). This is due to the strong transient PN input that overcomes high KC thresholds, whereas tonic PN input isn’t strong enough. In the absence of SFA in PNs, PNs show a strong constant population rate response. With constant PN input, KCs still show pronounced onset responses due to SFA. However, temporal sparseness is lower (cf. Fig 4-1A), because of non-zero tonic KC firing rate.

Minor issues

------------

#4 page 4, receptor input: are real receptor responses not adapting and really Poisson? I am not an expert for olfactory systems, but in any other system receptor responses do adapt (to about 50 % of the initial peak firing rate) and strong responses are definitely not Poisson like. Please motivate your choice.

The reviewer is of course right, olfactory receptor neurons are primary sensory neurons and they do show adaptation as is the case in sensory neurons of other modalities. Compared to the auditory system of insects the adaptation in the insect OSNs has somewhat longer time constants. The output of the transduction process of receptor events has been claimed to be Poisson like such that the spike generation mechanism receives a shot-noise like input (Nagel & Wilson, 2011). The spiking output of the ORNs, however, appears to be adaptive (e.g. Nagel & Wilson, 2011).

Here, we made a simplified assumption of Poisson input for two reasons: 1) The Poisson input to PNs and LNs ensures that the effects in our network is a pure effect of adaptation in the AL and MB alone; 2) full conductance based network would involve some ∼2.000 ORNs and Poisson input to each of them. This makes full network simulation for network analyses difficult. This simplification thus renders network analyses for different parameters feasible.

#5 Eq 10, lines 197-200: What is your rational to put noise only into the adaptation current? This also implies that in your scenarios without adaptation, there is no noise source at all in the system. I would instead add white noise to the current equations 2 - 4.

The main source of variability in the system is the input noise due to the Poisson inputs. Therefore, there is variability in the spiking activity of all cell types, for all scenarios. The rationale for adding noise to the adaptation current is easily explained. In our model, adaptation currents are caused by spike-triggered adaptation. Due to temporally sparse firing patterns, the majority of KC adaptation currents builds up at the stimulus onset, and remains largely unperturbed during and after the stimulation because spontaneous KC firing is scarce. In contrast to the spiking activity, there is no adaptation current fluctuations without an intrinsic noise source. Without adaptation noise, KC activation patterns stored in the adaptation levels would permit decoding accuracy on unrealistic time scales because - although decaying - the relations between the current amplitudes remain fixed. While the adaptation levels in the KC population decay exponentially, the decoding analysis could recover stimulus identity from relative adaptation levels, even if the absolute currents decayed close to zero (data not shown).

We have rephrased and extended the sentence starting in line 452 to clarify the effect of adaptation noise on decoding.

Adding intrinsic noise to the conductances in all the current balance equations 2-4 is beyond the scope of the present MS and it should not alter the general results of our study.

#6 Tab 2, Eq 2-4: All three neuron types are modelled with the same parameter (membrane time constants, leak, threshold, etc). Can you cite some literature to motivate this?

Our motivation was to define a generic model of the insect olfactory pathway. This model should establish the most basic bauplan of the insect olfactory pathway that is homologous across species, accommodating lateral inhibition in the antennal lobe and cellular adaptation. To focus on the effect of adaptation (strength) and lateral inhibition (strength) and to stress the generality of our results, we deliberately decided to use the same cell parameters across all neurons rather than using species- and neuron-type specific parameters. In the revised Methods section “Neuron Model”, we now made our reasoning for our choice of a single set of cell parameters explicit.

There is a second reason for using a single generic set of cell parameters. We have been gathering the available electrophysiological characterizations of specific neuron types across species from experimental literature and through direct communication with collaborating labs. Indeed, documentation of cell parameters based on scrutinized experiments is rather scarce. There is not a single species for which cell parameters have been characterized for all cell types and one would have to combine parameters from different species. More importantly, intracellular electrophysiological cell characterization from somatic recordings is particularly difficult in invertebrates. The somata are often tiny and distal from the spike initiation zones. Space clamp across wide axo-dendritic arborizations is thus hardly achievable and hence, estimated capacitances for instance apply only locally to the site of measurement. In KCs, for example, cell bodies are tiny (2∼3 mum) and distal from the axo-dendritic compartments and spike initiation zones. In cockroaches the measured somatic capacity is in the range of 2-3 pF, a value that is unrealistically small (personal communication Peter Kloppenburg) for the axo-dendritic compartments. An important physiological fact, however, is the existence of channels that mediate a very strong adaptation current in KCs of the cockroach (Demmer & Kloppenburg, 2009).

#7 page 7 simulation conditions: for the “no adaptation” scenario you replace the adaptation current by a constant one to ensure the right firing regime. For the “no lateral inhibition” scenario you scale synaptic weights instead. In this way you also modify temporal dynamics of input currents, in particular because tau_E < tau_I. Why not also just putting in compensatory currents?

We thank the reviewer for the valuable feedback on the compensation mechanisms. Indeed, we deliberately used two different compensation mechanisms in the model.

We have now revised the Methods section “Simulation Scenarios” to make the difference between the compensation mechanisms explicit.

The rationale is that adaptation is a cellular mechanism (modelled as a current) and therefore replaced by a compensatory current. Lateral inhibition is a network effect, conveyed by synaptic transmission, and therefore compensated by scaling of synaptic weights. With the “no lateral inhibition” scenario we provide a control condition, which deliberately does not include slow inhibitory synaptic dynamics. In addition scaling of weights provides compensation during spontaneous as well as evoked activity, whereas a compensatory current would be dependent on the input.

#8 Fig 1 A: Add arrows to the lateral inhibition

We have now added symbols in Fig 1A to indicate the synapses between LNs and PNs.

#9 Fig 2 B also indicate that x-axis is time

We have now indicated the time axis in Fig 2B.

#10 Fig 2: Add panels showing firing rate averaged over some time interval

as a function of PN neuron # and discuss these in the text.

We have now added supporting Figure 2-1 showing PN spike counts obtained during one second of stimulation for all four scenarios. We now discuss the sharpening of the PN response profile in line 592.

#11 Fig 4 B: alpha=0 population sparseness: the values should depend on some of your model parameter (in particular PN-KC connections). Can you elaborate on this?

This minor issue relates to major issue 1 (cf. above for additional analyses). KC sparseness does depend on the PN-KC connection probability - the smaller the average number of PNs connected to a single KC (indegree) for otherwise fixed KC cell parameters, the lower will be the response probability of KCs for any given stimulus and the higher will be the effective population sparseness. The number of 12 PNs per KC lies within the range of experimental estimates, we have now added additional references on experimental quantification PN-KC connections and provide the estimated numbers for k in the text (Discussion). Naturally, also the excitability of KCs regulates whether and how strong a KC responds to a given amount of input. Excitability in our model is related to the weight of the KC input synapses, the leak conductance and the threshold. We keep these parameters fix.

#12 Line 307 & 421: Can you specify what is quantitatively similar to the experimental data? Mean firing rates? Adaptation time constants? etc.

Lines 307f and 421f (in the originally submitted version) refer to an agreement of the sparseness measure S applied to population sparseness and temporal sparseness. In the revised MS we now provide quantitative experimental estimates for comparison in the Results section:

- Number of spikes per KC in response to a stimulus in (lines 335 ff). Experimental estimates lie in the range of 1-5 spikes (Turner et al., 2008; Ito et al., 2008). This matches to our simulation results of 1-3 spikes (Fig 6).

- Fraction of stimulus-activated KCs in (lines 344 ff). The experimentally estimated fraction of activated KCs, which lies in the range of 5-10% measured in the fruit fly (Honegger et al., 2011; Turner et al., 2008) and 6-11% in the locust (Perez-Orive et al., 2002; Broome et al., 2006). This matches our observation of average 9% activated KCs (Fig. 3 and Fig. 6-1). The experimentally measured range is now also provided in the caption of supplemental Figure 6-1 for comparison with our data.

- Experimentally reported values for population sparseness in (lines 344 ff). Perez-Orive et al. (2002) estimated the population sparseness in the locust as S=0.9. Ito et al. (2008) estimated S=0.79 in extracellular recordings from the locust. This again matches well with our quantitative results (Fig. 4B and Fig. 4-1).

#13 Fig 5 A bottom & line 316-317: I can not really follow your statements given this example: From the 5 white dots in the A&B case 4 are from B and only one from A, whereas two from A seem to be shared by B.

Our statement in the referenced lines (line 371) now reads “KCs responding exclusively to odor A or odor B are shown in gray, whereas KCs responding to both odors are color coded. With lateral inhibition, most of the KC responses were unique to odor A or odor B (shown in gray in Fig. 5B) and only relatively few KCs were activated by both odors.” This is correct. Fig. 5B shows the overlap of the two individual responses that are shown in Fig. 5A. There are a number of gray (not white) entries. These indicate KCs that did respond only to one of the two odors A or B. The hot color entries in the KC matrix indicate those KCs that responded to both odors, the color code indicates the number of response spikes, typically between 1 and 2, max. 3 spikes. There are clearly more gray matrix entries than hot color entries, i.e. the majority of KCs responded exclusively to one of the two odors.

#14 Line 366: no it is not the adaptation noise it is simply the adaptation current that is increased by the spike but that decays with a slow time constant and in this way carries information about previous spikes into the future.

This issue is related to and explained in the answer to the minor issue #5 above. We have rephrased and extended the sentence starting in line 366 to clarify the effect of adaptation noise.

#15 Discussion lines 423-435 and 446-454: how exactly do all these experimental findings relate to your model. Is forward inhibition different from SFA? In what ways? Are your conclusion really very general or are there other possibilities that would change the picture? Please really discuss these issues.

In lines 423-435 we had referred to feedback inhibition, not to feed-forward inhibition. We have now thoroughly revised the Discussion on population sparseness and the role of lateral inhibition as compared to feedback inhibition and the sparse connectivity scheme that provides the structural basis for a sparse code . As explained, feedback inhibition from the mushroom body output onto the MB input (acting on the synaptic densities that connect PNs and KCs) has been established in various insect species. It has been shown in Drosophila that specifically blocking this feedback inhibition reduces population sparseness. We believe that the mechanism that we propose (lateral inhibition) and feedback inhibition from the MB output act in concert and might be regulated independently. In particular, lateral inhibition could be modulated through neuromodulatory input, this aspect has now entered the revised discussion. We are confident that the revised and extended discussion provides more clarity.

#16 Discussion line 474ff: simply explain that the calcium flows in because of the (temporal sparse) spikes. Because calcium is removed from the intracellular space only with a slow time constant the information of the spikes is still there after quite a while.

Thank you for pointing out this missing explanation. We have now rephrased the relevant section (lines 634-638) in the discussion. In addition, we have now motivated the phenomenological SFA model in the method section.

Reviewer 2:

This manuscript is well written and systematically analyzes the contribution of spike frequency adaptation and lateral inhibition to the temporal and population sparseness of population responses in the olfactory system.

We thank the reviewer for her/his positive evaluation of the present work. We address all comments point-to-point.

There a number of minor comments that need to be addressed:

Line 100, not clear sentence that “simulations were initialized for 2000 ms without recording the activity” Please clarify how the simulations were initialized.

We now have clarified in the text (lines 103-105): the network was pre-run for 2000 ms without recording the activity in order to achieve steady state conditions (warm up).

In Eq. 5 please clarify how spikes were generated, was it using Poisson process with rate from Eq. 1?

Poisson spike trains were approximated by a Bernoulli process generated by the software brian simulator. Time step was set to 0.1 ms. Indeed we used firing rates (Poisson intensities) as given by Eq 1.

Lines 313-314, the sentence that starts “Given sparse population responses” needs to be rephrased.

We have rephrased the sentence as follows: “Does an increased KC population sparseness lead to less overlap between MB odor representations?”

Lines 345, the information about the stimulus identity has to be present in AL and MB representation. Maybe rephrase to state such as “The data were analyzed to determine the time course with which stimulus identity could be decoded from AL and MB responses”

We have rephrased line 345 as follows “The data were analyzed to determine the time course with which stimulus identity could be decoded from AL and MB responses”

Lines 387, double period.

We have cleared the double period from the text.

## References

[B1] Albus JS (1971) A theory of cerebellar function. Math Biosci 10:25–61. 10.1016/0025-5564(71)90051-4

[B2] Ardin P, Peng F, Mangan M, Lagogiannis K, Webb B (2016) Using an insect mushroom body circuit to encode route memory in complex natural environments. PLoS Comput Biol 12:e1004683 10.1371/journal.pcbi.1004683 26866692 PMC4750948

[B3] Assisi C, Stopfer M, Laurent G, Bazhenov M (2007) Adaptive regulation of sparseness by feedforward inhibition. Nat Neurosci 10:1176–1184. 10.1038/nn1947 17660812 PMC4061731

[B4] Barlow H (1961) Possible principles underlying the transformations of sensory messages In: Sensory communication: contributions to the symposium on principles of sensory communication, July 19 – August 1, 1959, Edicott House, M.I.T., Vol 6 (Rosenblith W, ed), pp 57–58. New York: Wiley.

[B5] Barlow H (2001) Redundancy reduction revisited. Network 12:241–253. 10.1080/net.12.3.241.253 11563528

[B6] Belmabrouk H, Nowotny T, Rospars J-P, Martinez D (2011) Interaction of cellular and network mechanisms for efficient pheromone coding in moths. Proc Natl Acad Sci U S A 108:19790–19795. 10.1073/pnas.1112367108 22109556 PMC3241803

[B7] Benda J, Herz AVM (2003) A universal model for spike-frequency adaptation. Neural Comput 15:2523–2564. 10.1162/089976603322385063 14577853

[B8] Bhandawat V, Olsen SR, Gouwens NW, Schlief ML, Wilson RI (2007) Sensory processing in the Drosophila antennal lobe increases reliability and separability of ensemble odor representations. Nat Neurosci 10:1474–1482. 10.1038/nn1976 17922008 PMC2838615

[B9] Broome B, Jayaraman V, Laurent G (2006) Encoding and decoding of overlapping odor sequences. Neuron 51:467–482. 10.1016/j.neuron.2006.07.018 16908412

[B10] Budick SA (2006) Free-flight responses of Drosophila melanogaster to attractive odors. J Exp Biol 209:3001–3017. 10.1242/jeb.02305 16857884

[B11] Buonomano DV, Maass W (2009) State-dependent computations: spatiotemporal processing in cortical networks. Nat Rev Neurosci 10:113–125. 10.1038/nrn2558 19145235

[B12] Campbell RAA, Honegger KS, Qin H, Li W, Demir E, Turner GC (2013) Imaging a population code for odor identity in the *Drosophila* mushroom body. J Neurosci 33:10568–10581. 10.1523/JNEUROSCI.0682-12.2013 23785169 PMC3685844

[B13] Capurro A, Baroni F, Olsson SB, Kuebler LS, Karout S, Hansson BS, Pearce TC (2012) Non-linear blend coding in the moth antennal lobe emerges from random glomerular networks. Front Neuroeng 5:6. 10.3389/fneng.2012.00006 22529799 PMC3329896

[B14] Caron SJC, Ruta V, Abbott LF, Axel R (2013) Random convergence of olfactory inputs in the Drosophila mushroom body. Nature 497:113–117. 10.1038/nature12063 23615618 PMC4148081

[B15] Celani A, Villermaux E, Vergassola M (2014) Odor landscapes in turbulent environments. Phys Rev X 4:041015 10.1103/PhysRevX.4.041015

[B16] Chou Y-H, Spletter ML, Yaksi E, Leong JCS, Wilson RI, Luo L (2010) Diversity and wiring variability of olfactory local interneurons in the Drosophila antennal lobe. Nat Neurosci 13:439–449. 10.1038/nn.2489 20139975 PMC2847188

[B17] Deisig N, Giurfa M, Sandoz JC (2010) Antennal lobe processing increases separability of odor mixture representations in the honeybee. J Neurophysiol 103:2185–2194. 10.1152/jn.00342.2009 20181736

[B18] Demmer H, Kloppenburg P (2009) Intrinsic membrane properties and inhibitory synaptic input of kenyon cells as mechanisms for sparse coding? J Neurophysiol 102:1538–1550. 10.1152/jn.00183.2009 19553491

[B19] Dylla KV, Galili DS, Szyszka P, Lüdke A (2013) Trace conditioning in insects-keep the trace. Front Physiol 4:67–12. 10.3389/fphys.2013.00067 23986710 PMC3750952

[B20] Egea-Weiss A, Renner A, Kleineidam CJ, Szyszka P (2018) High precision of spike timing across olfactory receptor neurons allows rapid odor coding in Drosophila. iScience 4:76–83. 10.1016/j.isci.2018.05.009 30240755 PMC6147046

[B21] Ermentrout B (1998) Linearization of F-I curves by adaptation. Neural Comput 10:1721–1729. 10.1162/089976698300017106 9744894

[B22] Faisal AA, Selen LPJ, Wolpert DM (2008) Noise in the nervous system. Nat Rev Neurosci 9:292–303. 10.1038/nrn2258 18319728 PMC2631351

[B23] Farkhooi F, Muller E, Nawrot M (2011) Adaptation reduces variability of the neuronal population code. Phys Rev E Stat Nonlin Soft Matter Phys 83:050905 10.1103/PhysRevE.83.050905 21728481

[B24] Farkhooi F, Froese A, Muller E, Menzel R, Nawrot MP (2013) Cellular adaptation facilitates sparse and reliable coding in sensory pathways. PLoS Comput Biol 9:e1003251. 10.1371/journal.pcbi.1003251 24098101 PMC3789775

[B25] Filla I, Menzel R (2015) Mushroom body extrinsic neurons in the honeybee (Apis mellifera) brain integrate context and cue values upon attentional stimulus selection. J Neurophysiol 114:2005–2014. 10.1152/jn.00776.2014 26224779 PMC4579295

[B26] Fisch K, Schwalger T, Lindner B, Herz A, Benda J (2012) Channel noise from both slow adaptation currents and fast currents is required to explain spike-response variability in a sensory neuron. J Neurosci 32:17332–17344. 10.1523/JNEUROSCI.6231-11.2012 23197724 PMC6621841

[B27] Friedrich RW, Wiechert MT (2014) Neuronal circuits and computations: pattern decorrelation in the olfactory bulb. FEBS Lett 588:2504–2513. 10.1016/j.febslet.2014.05.055 24911205

[B28] Froese A, Szyszka P, Menzel R (2014) Effect of GABAergic inhibition on odorant concentration coding in mushroom body intrinsic neurons of the honeybee. J Comp Physiol A Neuroethol Sens Neural Behav Physiol 200:183–195. 10.1007/s00359-013-0877-8 24362942

[B29] Fuchs JL, Brown PB (1984) Two-point discriminability: relation to properties of the somatosensory system. Somatosens Res 2:163–169. 10.1080/07367244.1984.11800556 6528150

[B30] Galili DS, Lüdke A, Galizia CG, Szyszka P, Tanimoto H (2011) Olfactory trace conditioning in *Drosophila*. J Neurosci 31:7240–7248. 10.1523/JNEUROSCI.6667-10.2011 21593308 PMC6622595

[B31] Galizia CG (2014) Olfactory coding in the insect brain: data and conjectures. Eur J Neurosci 39:1784–1795. 10.1111/ejn.12558 24698302 PMC4237541

[B32] Goodman DFM, Brette R (2009) The Brian simulator. Front Neurosci 3:192–197. 10.3389/neuro.01.026.2009 20011141 PMC2751620

[B33] Grünewald B (1999) Morphology of feedback neurons in the mushroom body of the honey bee, Apis mellifera. J Comp Neurol 404:114–126. 9886029 10.1002/(sici)1096-9861(19990201)404:1<114::aid-cne9>3.3.co;2-r

[B34] Gupta N, Stopfer M (2012) Functional analysis of a higher olfactory center, the lateral horn. J Neurosci 32:8138–8148. 10.1523/JNEUROSCI.1066-12.2012 22699895 PMC3391592

[B35] Haehnel M, Menzel R (2010) Sensory representation and learning-related plasticity in mushroom body extrinsic feedback neurons of the protocerebral tract. Front Syst Neurosci 4:161. 10.3389/fnsys.2010.00161 21212833 PMC3014600

[B36] Haenicke J, Yamagata N, Zwaka H, Nawrot M, Menzel R (2018) Neural correlates of odor learning in the presynaptic microglomerular circuitry in the honeybee mushroom body calyx. eNeuro 5:ENEURO.0128-18.2018 10.1523/ENEURO.0128-18.2018 PMC6011417 29938214

[B37] Haider B, Krause MR, Duque A, Yu Y, Touryan J, Mazer JA, McCormick DA (2010) Synaptic and network mechanisms of sparse and reliable visual cortical activity during nonclassical receptive field stimulation. Neuron 65:107–121. 10.1016/j.neuron.2009.12.005 20152117 PMC3110675

[B38] Hartline HK, Wagner HG, Ratliff F (1956) Inhibition in the eye of limulus. J Gen Physiol 39:651–673. 10.1085/jgp.39.5.651 13319654 PMC2147566

[B39] Hildebrandt KJ, Ronacher B, Hennig RM, Benda J (2015) A neural mechanism for time-window separation resolves ambiguity of adaptive coding. PLoS Biol 13:e1002096 10.1371/journal.pbio.1002096 25761097 PMC4356587

[B40] Honegger KS, Campbell RAA, Turner GC (2011) Cellular-resolution population imaging reveals robust sparse coding in the *Drosophila* mushroom body. J Neurosci 31:11772–11785. 10.1523/JNEUROSCI.1099-11.2011 21849538 PMC3180869

[B41] Hromádka T, DeWeese MR, Zador AM (2008) Sparse representation of sounds in the unanesthetized auditory cortex. PLoS Biol 6:e16. 10.1371/journal.pbio.0060016 18232737 PMC2214813

[B42] Huerta R, Nowotny T (2009) Fast and robust learning by reinforcement signals: explorations in the insect brain. Neural Comput 21:2123–2151. 10.1162/neco.2009.03-08-733 19538091

[B43] Isaacson JS (2010) Odor representations in mammalian cortical circuits. Curr Opin Neurobiol 20:328–331. 10.1016/j.conb.2010.02.004 20207132 PMC2896888

[B44] Ito I, Ong RC-Y, Raman B, Stopfer M (2008) Sparse odor representation and olfactory learning. Nat Neurosci 11:1177–1184. 10.1038/nn.2192 18794840 PMC3124899

[B45] Jortner RA, Farivar SS, Laurent G (2007) A simple connectivity scheme for sparse coding in an olfactory system. J Neurosci 27:1659–1669. 10.1523/JNEUROSCI.4171-06.2007 17301174 PMC6673743

[B46] Kanerva P (1988) Sparse distributed memory. Cambridge, MA: MIT.

[B47] Kee T, Sanda P, Gupta N, Stopfer M, Bazhenov M (2015) Feed-forward versus feedback inhibition in a basic olfactory circuit. PLOS Comput Biol 11:e1004531 10.1371/journal.pcbi.1004531 26458212 PMC4601731

[B48] Kloppenburg P, Nawrot MP (2014) Neural coding: sparse but on time. Curr Biol 24:R957–R959. 10.1016/j.cub.2014.08.041 25291636

[B49] Kremer MC, Christiansen F, Leiss F, Paehler M, Knapek S, Andlauer TF, Förstner F, Kloppenburg P, Sigrist SJ, Tavosanis G (2010) Structural long-term changes at mushroom body input synapses. Curr Biol 20:1938–1944. 10.1016/j.cub.2010.09.060 20951043

[B50] Krofczik S, Menzel R, Nawrot MP (2009) Rapid odor processing in the honeybee antennal lobe network. Front Comput Neurosci 2:9. 10.3389/neuro.10.009.2008 19221584 PMC2636688

[B51] Kuffler SW (1953) Discharge patterns and functional organization of mammalian retina. J Neurophysiol 16:37–68. 10.1152/jn.1953.16.1.37 13035466

[B52] Laughlin SB, Sejnowski TJ (2003) Communication in neuronal networks. Science 301:1870–1874. 10.1126/science.1089662 14512617 PMC2930149

[B53] Laurent G (2002) Olfactory network dynamics and the coding of multidimensional signals. Nat Rev Neurosci 3:884–895. 10.1038/nrn964 12415296

[B54] Lei Z, Chen K, Li H, Liu H, Guo A (2013) The GABA system regulates the sparse coding of odors in the mushroom bodies of Drosophila. Biochem Biophys Res Commun 436:35–40. 10.1016/j.bbrc.2013.05.036 23707718

[B55] Leiss F, Groh C, Butcher NJ, Meinertzhagen IA, Tavosanis G (2009) Synaptic organization in the adult Drosophila mushroom body calyx. J Comp Neurol 517:808–824. 10.1002/cne.22184 19844895

[B56] Lin AC, Bygrave AM, de Calignon A, Lee T, Miesenböck G (2014) Sparse, decorrelated odor coding in the mushroom body enhances learned odor discrimination. Nat Neurosci 17:559–568. 10.1038/nn.3660 24561998 PMC4000970

[B57] Liu X, Davis RL (2009) The GABAergic anterior paired lateral neuron suppresses and is suppressed by olfactory learning. Nat Neurosci 12:53–59. 10.1038/nn.2235 19043409 PMC2680707

[B58] Lizbinski KM, Dacks AM (2018) Intrinsic and extrinsic neuromodulation of olfactory processing. Front Cell Neurosci 11:424 10.3389/fncel.2017.00424 29375314 PMC5767172

[B59] Lüdke A, Raiser G, Nehrkorn J, Herz AVM, Galizia CG, Szyszka P (2018) Calcium in Kenyon cell somata as a substrate for an olfactory sensory memory in Drosophila. Front Cell Neurosci 12:128 10.3389/fncel.2018.00197 29867361 PMC5960692

[B60] Lundstrom BN, Higgs MH, Spain WJ, Fairhall AL (2008) Fractional differentiation by neocortical pyramidal neurons Nature neuroscience 11:1335. 18931665 10.1038/nn.2212 PMC2596753

[B61] Luo SX, Axel R, Abbott LF (2010) Generating sparse and selective third-order responses in the olfactory system of the fly. Proc Natl Acad Sci U S A 107:10713–10718. 10.1073/pnas.1005635107 20498080 PMC2890779

[B62] Marr BYD (1969) A theory of cerebellar cortex. J Physiol 202:437–470. 10.1113/jphysiol.1969.sp008820 5784296 PMC1351491

[B63] Mazor O, Laurent G (2005) Transient dynamics versus fixed points in odor representations by locust antennal lobe projection neurons. Neuron 48:661–673. 10.1016/j.neuron.2005.09.032 16301181

[B64] Meyer A, Galizia CG, Nawrot MP (2013) Local interneurons and projection neurons in the antennal lobe from a spiking point of view. J Neurophysiol 110:2465–2474. 10.1152/jn.00260.2013 24004530

[B65] Mosqueiro TS, Huerta R (2014) Computational models to understand decision making and pattern recognition in the insect brain. Curr Opin Insect Sci 6:80–85. 10.1016/j.cois.2014.10.005 25593793 PMC4289906

[B66] Müller J, Nawrot M, Menzel R, Landgraf T (2018) A neural network model for familiarity and context learning during honeybee foraging flights. Biol Cybern 112:113–126. 10.1007/s00422-017-0732-z 28917001

[B67] Nagel KI, Wilson RI (2011) Biophysical mechanisms underlying olfactory receptor neuron dynamics. Nat Neurosci 14:208–216. 10.1038/nn.2725 21217763 PMC3030680

[B68] Nawrot MP (2012) Dynamics of sensory processing in the dual olfactory pathway of the honeybee. Apidologie 43:269–291. 10.1007/s13592-012-0131-3

[B69] Nowotny T, Huerta R, Abarbanel HD, Rabinovich MI (2005) Self-organization in the olfactory system: one shot odor recognition in insects. Biol Cybern 93:436–446. 10.1007/s00422-005-0019-7 16320081

[B70] Olsen SR, Wilson RI (2008) Lateral presynaptic inhibition mediates gain control in an olfactory circuit. Nature 452:956–960. 10.1038/nature06864 18344978 PMC2824883

[B71] Olsen SR, Bhandawat V, Wilson RI (2010) Divisive normalization in olfactory population codes. Neuron 66:287–299. 10.1016/j.neuron.2010.04.009 20435004 PMC2866644

[B72] Oswald A-MM, Schiff ML, Reyes AD (2006) Synaptic mechanisms underlying auditory processing. Curr Opin Neurobiol 16:371–376. 10.1016/j.conb.2006.06.015 16842988

[B73] Papadopoulou M, Cassenaer S, Nowotny T, Laurent G (2011) Normalization for sparse encoding of odors by a wide-field interneuron. Science 332:721–725. 10.1126/science.1201835 21551062 PMC3242050

[B74] Pedregosa F, Varoquaux G, Gramfort A, Michel V, Thirion B, Grisel O, Blondel M, Prettenhofer P, Weiss R, Dubourg V, Vanderplas J, Passos A, Cournapeau D, Brucher M, Perrot M, Duchesnay É (2012) Scikit-learn: machine learning in Python. J Mach Learn Res 12:2825–2830.

[B75] Peng F, Chittka L (2016) A simple computational model of the bee mushroom body can explain seemingly complex forms of olfactory learning and memory. Curr Biol 0:2597–2604. 10.1016/j.cub.2017.05.037 28017607

[B76] Perez-Orive J, Mazor O, Turner GC, Cassenaer S, Wilson RI, Laurent G (2002) Oscillations and sparsening of odor representations in the mushroom body. Science 297:359–365. 10.1126/science.1070502 12130775

[B77] Perisse E, Waddell S (2011) Associative memory: without a trace. Curr Biol 21:R579–R581. 10.1016/j.cub.2011.06.012 21820619

[B78] Rish I (2000) An empirical study of the naive Bayes classifier. Paper presented at ECAI-2000 Workshop: Empirical Methods in Artificial Intelligence, Berlin, August.

[B79] Schmuker M, Yamagata N, Nawrot M, Menzel R (2011) Parallel representation of stimulus identity and intensity in a dual pathway model inspired by the olfactory system of the honeybee. Frontiers in neuroengineering 4:17. 22232601 10.3389/fneng.2011.00017 PMC3246696

[B80] Schmuker M, Pfeil T, Nawrot MP (2014) A neuromorphic network for generic multivariate data classification. Proc Natl Acad Sci U S A 111:2081–2086. 10.1073/pnas.1303053111 24469794 PMC3926020

[B81] Schwalger T, Fisch K, Benda J, Lindner B (2010) How noisy adaptation of neurons shapes interspike interval histograms and correlations. PLoS Comput Biol 6:e1001026. 10.1371/journal.pcbi.1001026 21187900 PMC3002986

[B82] Stierle JS, Galizia CG, Szyszka P (2013) Millisecond stimulus onset-asynchrony enhances information about components in an odor mixture. J Neurosci 33:6060–6069. 10.1523/JNEUROSCI.5838-12.2013 23554487 PMC6618935

[B83] Stopfer M, Jayaraman V, Laurent G (2003) Intensity versus identity coding in an olfactory system. Neuron 39:991–1004. 10.1016/j.neuron.2003.08.011 12971898

[B84] Strube-Bloss MF, Herrera-Valdez M. a, Smith BH (2012) Ensemble response in mushroom body output neurons of the honey bee outpaces spatiotemporal odor processing two synapses earlier in the antennal lobe. PLoS One 7:e50322. 10.1371/journal.pone.0050322 23209711 PMC3510213

[B85] Szyszka P, Ditzen M, Galkin A, Galizia CG, Menzel R, Ditzen M, Galkin A, Giovanni C (2005) Sparsening and temporal sharpening of olfactory representations in the honeybee mushroom bodies. J Neurophysiol 94:3303–3313. 10.1152/jn.00397.2005 16014792

[B86] Szyszka P, Demmler C, Oemisch M, Sommer L, Biergans S, Birnbach B, Silbering AF, Galizia CG (2011) Mind the gap: olfactory trace conditioning in honeybees. J Neurosci 31:7229–7239. 10.1523/JNEUROSCI.6668-10.2011 21593307 PMC6622586

[B87] Szyszka P, Stierle JS, Biergans S, Galizia CG (2012) The speed of smell: odor-object segregation within milliseconds. PLoS One 7:e36096. 10.1371/journal.pone.0036096 22558344 PMC3338635

[B88] Szyszka P, Gerkin RC, Galizia CG, Smith BH (2014) High-speed odor transduction and pulse tracking by insect olfactory receptor neurons. Proc Natl Acad Sci U S A 111:16925–16930. 10.1073/pnas.1412051111 25385618 PMC4250155

[B89] Takahashi N, Katoh K, Watanabe H, Nakayama Y, Iwasaki M, Mizunami M, Nishino H (2017) Complete identification of four giant interneurons supplying mushroom body calyces in the cockroach Periplaneta americana. J Comp Neurol 525:204–230. 10.1002/cne.24108 27573362

[B90] Treves A (1993) Mean-field analysis of neuronal spike dynamics. Network 4:259–284. 10.1088/0954-898X_4_3_002

[B91] Treves A, Rolls ET (1991) What determines the capacity of autoassociative memories in the brain? Network 2:371–397. 10.1088/0954-898X_2_4_004

[B92] Tripp BP, Eliasmith C (2010) Population models of temporal differentiation. Neural computation 22:621–659. 19922294 10.1162/neco.2009.02-09-970

[B93] Turner GC, Bazhenov M, Laurent G (2008) Olfactory representations by Drosophila mushroom body neurons. J Neurophysiol 99:734–746. 10.1152/jn.01283.2007 18094099

[B94] Uchida N, Poo C, Haddad R (2013) Coding and transformations in the olfactory system. Annu Rev Neurosci 37:363–385. 10.1146/annurev-neuro-071013-013941 24905594

[B95] Van Breugel F, Dickinson MH (2014) Plume-tracking behavior of flying Drosophila emerges from a set of distinct sensory-motor reflexes. Curr Biol 24:274–286. 10.1016/j.cub.2013.12.023 24440395

[B96] Vickers NJ (2000) Mechanisms of animal navigation in odor plumes. Biol Bull 198:203–212. 10786941 10.2307/1542524

[B97] Vickers NJ, Christensen TA, Baker TC, Hildebrand JG (2001) Odour-plume dynamics influence file brain’s olfactory code. Nature 410:466–470. 10.1038/35068559 11260713

[B98] Vinje WE, Gallant JL (2000) Sparse coding and decorrelation in primary visual cortex during natural vision. Science 287:1273–1276. 10.1126/science.287.5456.1273 10678835

[B99] Wessnitzer J, Young JM, Armstrong JD, Webb B (2012) A model of non-elemental olfactory learning in Drosophila. J Comput Neurosci 32:197–212. 10.1007/s10827-011-0348-6 21698405

[B100] Willmore B, Tolhurst DJ (2001) Characterizing the sparseness of neural codes. Network 12:255–270. 11563529

[B101] Wilson RI (2013) Early olfactory processing in Drosophila: mechanisms and principles. Annu Rev Neurosci 36:217–241. 10.1146/annurev-neuro-062111-150533 23841839 PMC3933953

[B102] Wilson RI, Laurent G (2005) Role of GABAergic inhibition in shaping odor-evoked spatiotemporal patterns in the *Drosophila* antennal lobe. J Neurosci 25:9069–9079. 10.1523/JNEUROSCI.2070-05.2005 16207866 PMC6725763

[B103] Wilson RI, Turner GC, Laurent G (2004) Transformation of olfactory representations in the Drosophila antennal lobe. Science 303:366–370. 10.1126/science.1090782 14684826

[B104] Wolfe J, Houweling AR, Brecht M (2010) Sparse and powerful cortical spikes. Curr Opin Neurobiol 20:306–312. 10.1016/j.conb.2010.03.006 20400290

[B105] Wüstenberg DG, Boytcheva M, Grünewald B, Byrne JH, Menzel R, Baxter D. a (2004) Current- and voltage-clamp recordings and computer simulations of Kenyon cells in the honeybee. J Neurophysiol 92:2589–2603. 10.1152/jn.01259.2003 15190098

[B106] Yen S-C, Baker J, Gray C (2010) Heterogeneity in the responses of adjacent neurons to natural stimuli in Cat striate cortex (abstract). J Vis 7:326 10.1167/7.9.326 17079343

