# Supplementary figures and images for: Circuit and Cellular Mechanisms Facilitate the Transformation from Dense to Sparse Coding in the Insect Olfactory System
Source: eNeuro. 2020 Mar 27;7(2):ENEURO.0305-18.2020. doi: 10.1523/ENEURO.0305-18.2020 (PMC7294456; doi:10.1523/ENEURO.0305-18.2020)

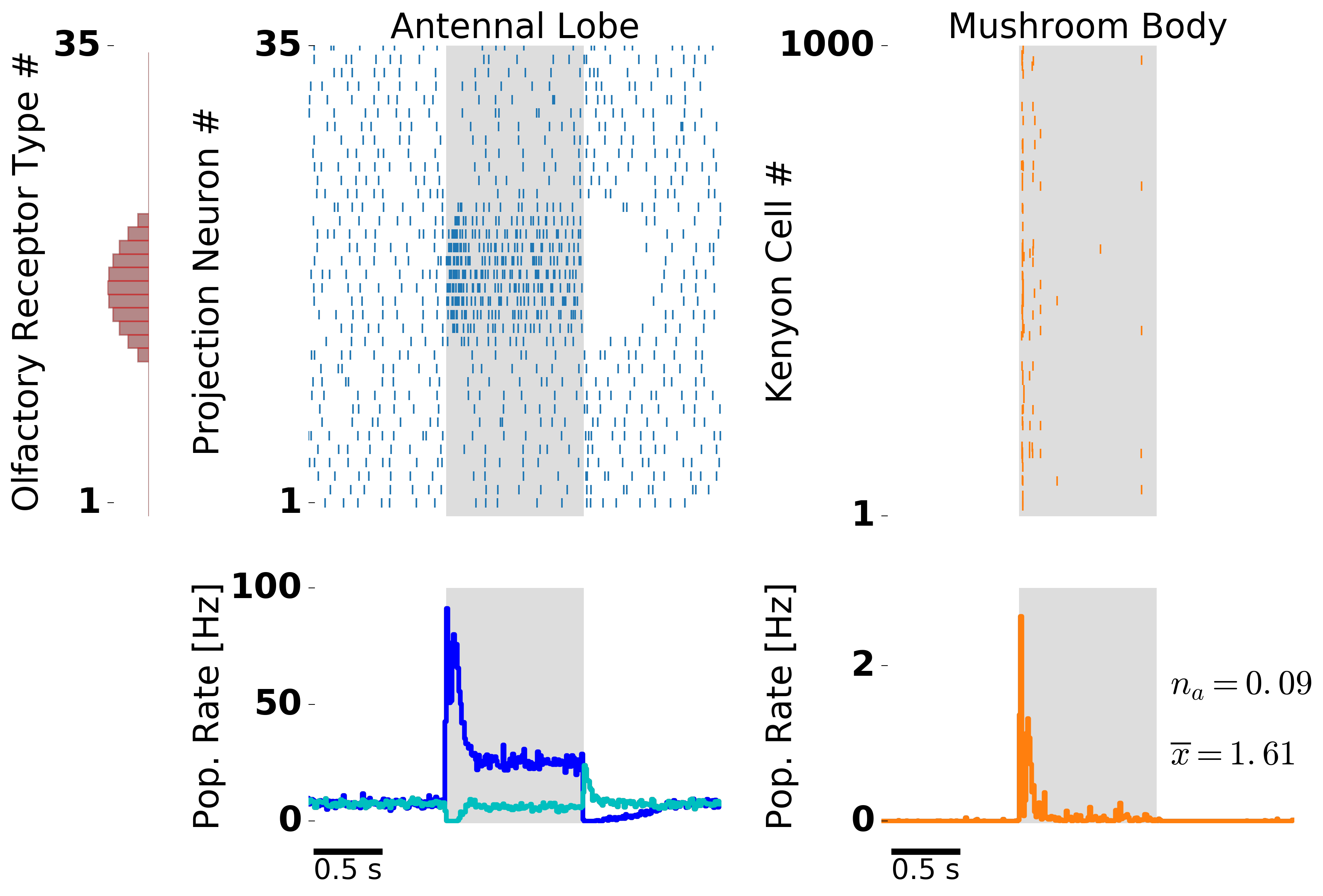

Supplement: Figure 1-1 — Odor response with selective adaptation in the LN and the PN population. Strong phasic PN input elicits phasic KC responses. High KC firing threshold ensures sparse responses in the absence of SFA in the KC population. Download Figure 1-1, TIF file. [file enu-eN-NWR-0305-18-s02.tif]

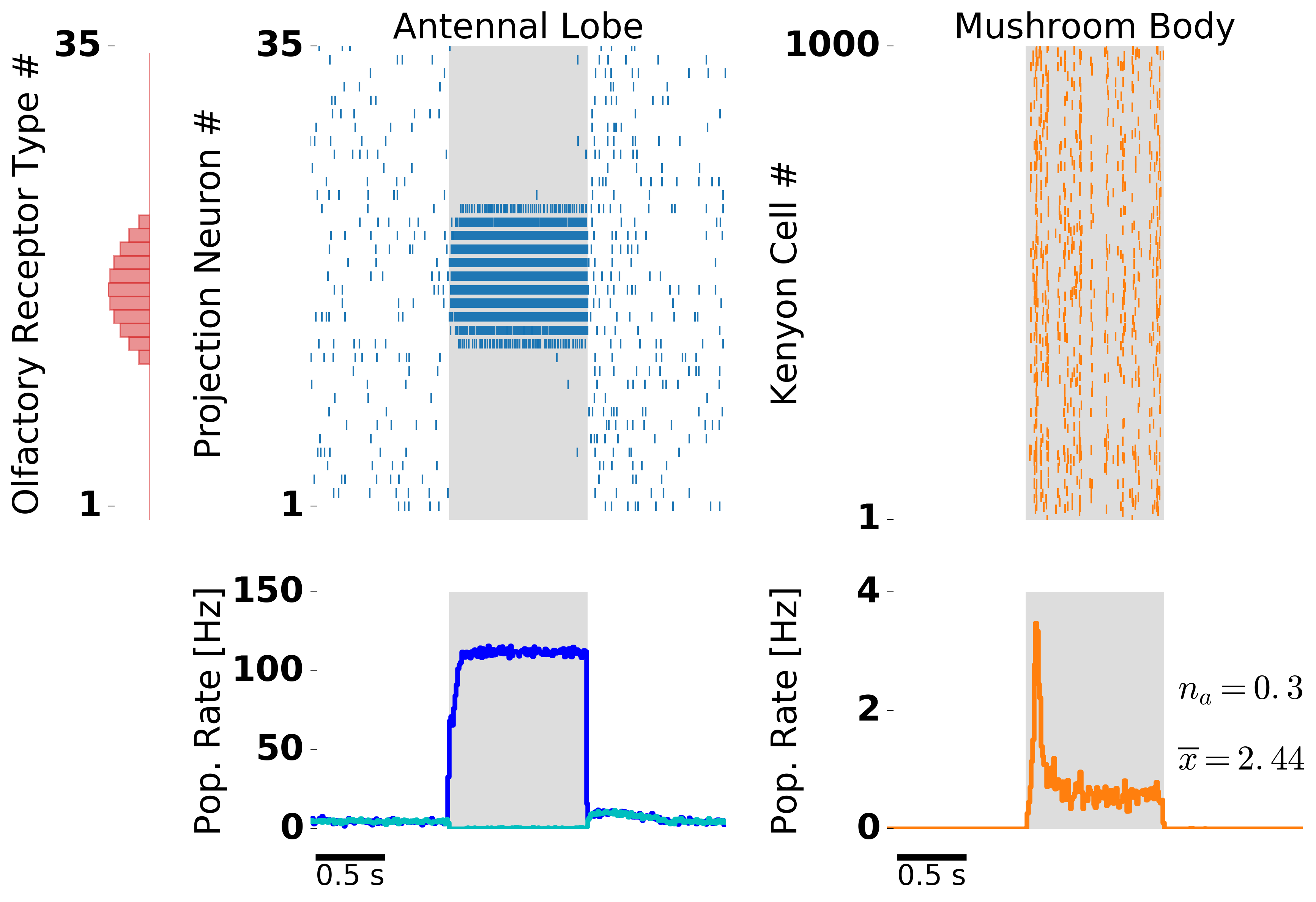

Supplement: Figure 1-2 — Odor response with selective adaptation in the LN and the KC population. The absence of SFA in the PN population was compensated by a constant current I0 = 0.38 nA. PNs show a constant population rate response with a slightly delayed onset due to inhibition by LNs. KCs show a strong onset population rate response and a nonzero tonic firing rate. Download Figure 1-2, TIF file. [file enu-eN-NWR-0305-18-s03.tif]

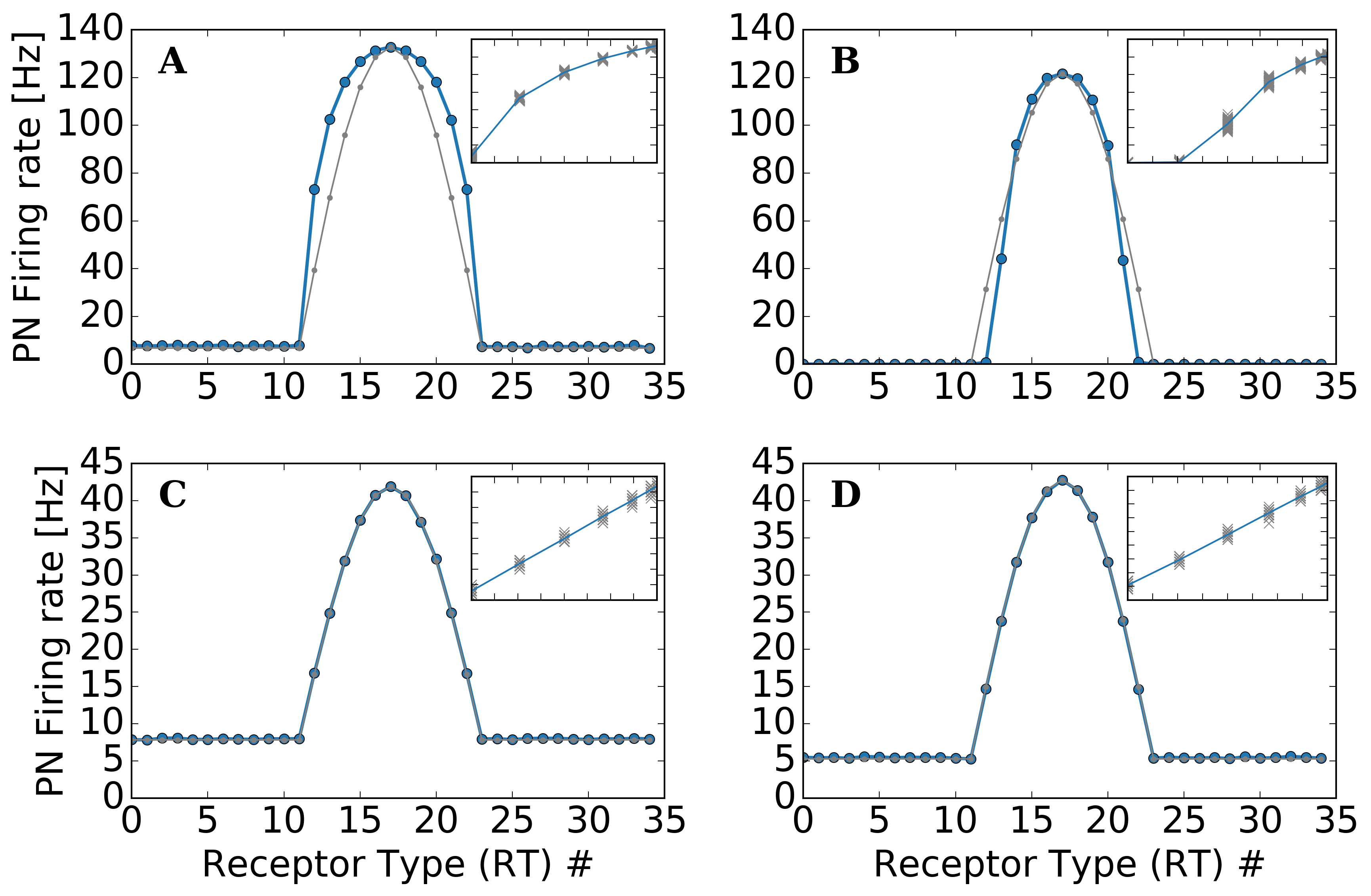

Supplement: Figure 2-1 — A, B, In the absence of adaptation (A, B), lateral inhibition (B) sharpens the PN tuning profile (blue). C, D, In the presence of adaptation, the PN tuning profile is not affected by lateral inhibition. The tuning profile was obtained by averaging PN firing rates during the 1 s stimulation window and across 50 trials. PNs receive input from ORNs of the corresponding type according to the receptor response profile. The receptor response profile (gray), rescaled between the minimum and maximum PN firing rates, is shown in all panels for comparison. The insets show the input–output relation between the ORN and the PN firing rates. Both averaged (blue line) and single-trial (gray crosses) PN firing rates are shown. Download Figure 2-1, TIF file. [file enu-eN-NWR-0305-18-s04.tif]

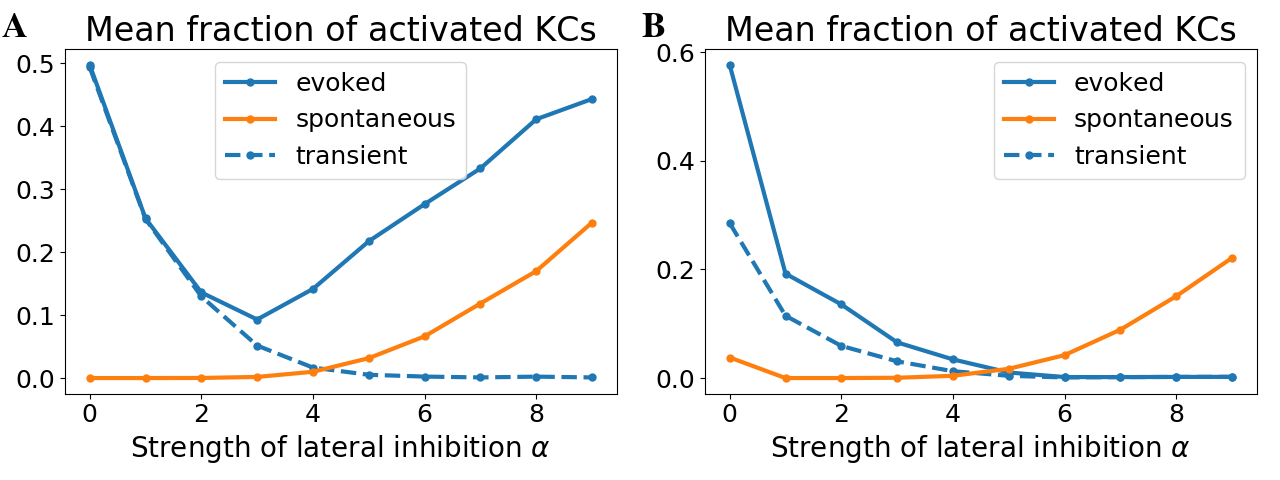

Supplement: Figure 6-1 — Mean fraction of activated KCs for different strengths of lateral inhibition. We obtained the fraction of activated KCs by counting KCs that have fired at least one spike during one of the given epochs: 1 s of stimulation, 1 s of spontaneous activity, and the first 50 ms after stimulus onset (transient response). A, In the presence of spike frequency adaptation the mean fraction of activated KCs during evoked activity (blue) shows a minimum for the intermediate strength of lateral inhibition. At the minimum, ∼10% of the KCs responded to the stimulus. This fits well with the experimentally reported values in the range of 5–11% (Turner et al., 2008; Honegger et al., 2011). B, In the absence of spike frequency adaptation, the mean fraction of activated KCs decreases with lateral inhibition during evoked activity (blue). Note that for α > 4 the fraction of responding KCs is close to zero or is zero. In the absence of spike frequency adaptation, and higher strengths of inhibition, KCs do not receive strong enough inputs to spike. Download Figure 6-1, TIF file. [file enu-eN-NWR-0305-18-s06.tif]

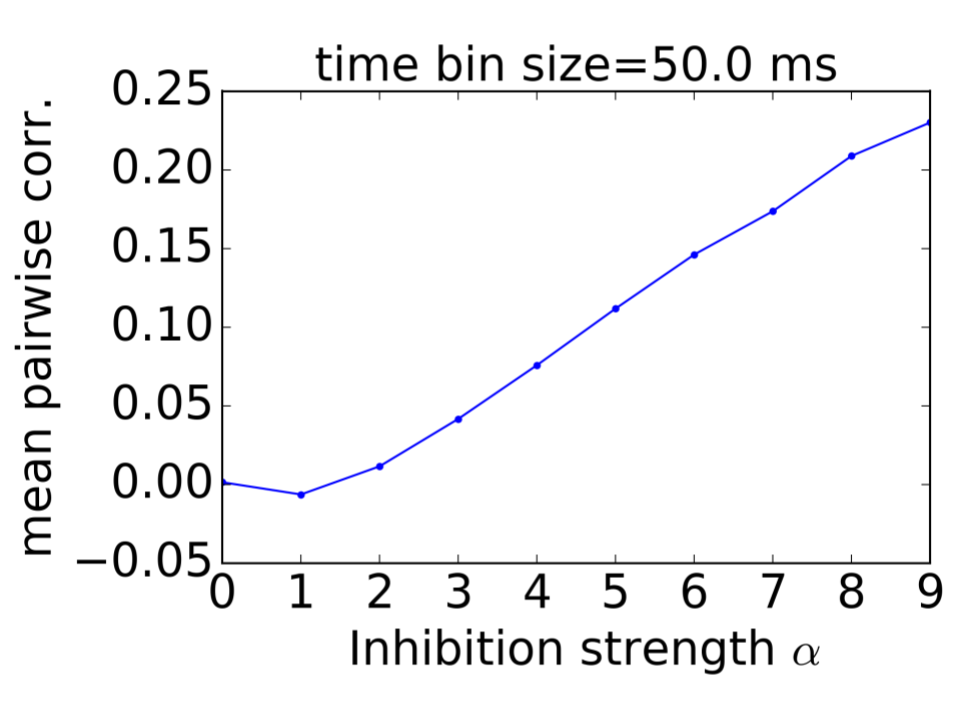

Supplement: Figure 6-2 — Mean pairwise PN cross-correlation for different strengths of lateral inhibition. For each PN, a vector obtained by binning the corresponding spike train into 50 ms windows was calculated. Pairwise correlation between the vectors was calculated and averaged over all PN pairs and 50 trials. Download Figure 6-2, TIF file. [file enu-eN-NWR-0305-18-s07.tif]
